# Excited scalar and pseudoscalar mesons in the extended linear sigma model

**DOI:** 10.1140/epjc/s10052-017-4962-y

**Published:** 2017-07-06

**Authors:** Denis Parganlija, Francesco Giacosa

**Affiliations:** 1 Institut für Theoretische Physik, Technische Universität Wien, Wiedner Hauptstr. 8-10, 1040 Vienna, Austria; 2 Institute of Physics, Jan Kochanowski University, ul. Swietokrzyska 15, 25-406 Kielce, Poland; 3 Institut für Theoretische Physik, Johann Wolfgang Goethe-Universität, Max-von-Laue-Str. 1, 60438 Frankfurt am Main, Germany

## Abstract

We present an in-depth study of masses and decays of excited scalar and pseudoscalar ${\bar{q}}q$ states in the Extended Linear Sigma Model (eLSM). The model also contains ground-state scalar, pseudoscalar, vector and axial-vector mesons. The main objective is to study the consequences of the hypothesis that the $f_0(1790)$ resonance, observed a decade ago by the BES Collaboration and recently by LHCb, represents an excited scalar quarkonium. In addition we also analyse the possibility that the new $a_0(1950)$ resonance, observed recently by BABAR, may also be an excited scalar state. Both hypotheses receive justification in our approach although there appears to be some tension between the simultaneous interpretation of $f_0(1790)$/$a_0(1950)$ and pseudoscalar mesons $\eta (1295)$, $\pi (1300)$, $\eta (1440)$ and *K*(1460) as excited ${\bar{q}}q$ states.

## Introduction

One of the most important features of strong interaction is the existence of the hadron spectrum. It emerges from confinement of quarks and gluons – degrees of freedom of the underlying theory, Quantum Chromodynamics (QCD) – in regions of sufficiently low energy where the QCD coupling is known to be large [1–4]. Although the exact mechanism of hadron formation in non-perturbartive QCD is not yet fully understood, an experimental fact is a very abundant spectrum of states possessing various quantum numbers, such as for example isospin *I*, total spin *J*, parity *P* and charge conjugation *C*.

This is in particular the case for the spectrum of mesons (hadrons with integer spin) that can be found in the listings of PDG – the Particle Data Group [5]. In the scalar channel ($J^{P}=0^{+}$), the following states are listed in the energy region up to approximately 2 GeV:

$$\begin{aligned}&f_{0}(500)/\sigma ,K_{0}^{\star }(800)/\kappa ,a_{0} (980),f_{0}(980),f_{0}(1370),\\&\quad K_{0}^{\star }(1430),a_{0}(1450),f_{0}(1500),f_{0}(1710), \\&\quad K_{0}^{\star }(1950),a_{0}(1950),f_{0}(2020), f_{0}(2100). \end{aligned}$$

The pseudoscalar channel ($J^{P}=0^{-}$) is similarly well populated:

$$\begin{aligned}&\pi ,K,\eta ,\eta ^{\prime }(958),\eta (1295),\pi (1300),\eta (1405),\\&\quad K(1460), \eta (1475),\eta (1760),\pi (1800),K(1830). \end{aligned}$$

A natural expectation founded in the Quark Model (see Refs. [6, 7]; for a modern and modified version see for example Refs. [8, 9]) is that the mentioned states can effectively be described in terms of constituent quarks and antiquarks – ground-state ${\bar{q}}q$ resonances. In this context, we define ground states as those with the lowest mass for a given set of quantum numbers *I*, *J*, *P* and *C*. Such a description is particularly successful for the lightest pseudoscalar states $\pi$, *K* and $\eta$.

However, this cannot be the full picture as the spectra contain more states than could be described in terms of the ground-state ${\bar{q}}q$ structure. A further natural expectation is then that the spectra may additionally contain first (radial) excitations of ${\bar{q}}q$ states, i.e., those with the same quantum numbers but with higher masses. (In the spectroscopic notation, the excited scalar and pseudoscalar states correspond, respectively, to the $2\, ^{3}P_0$ and $2\, ^{1}S_0$ configurations.) Of course, the possibility to study such states depends crucially on the identification of the ground states themselves; in the case of the scalar mesons, this is not as clear as for the pseudoscalars. Various hypotheses have been suggested for the scalar-meson structure, including meson–meson molecules, ${\bar{q}}{\bar{q}}qq$ states and glueballs, bound states of gluons – see, e.g., Refs. [10–76]. Results of these studies are at times conflicting but the general conclusion is nonetheless that the scalar ${\bar{q}}q$ ground states (as well as the glueball and the low-energy four-quark states) are well defined and positioned in the spectrum of particles up to and including the $f_0(1710)$ resonance.

The main objective of this work is then to ascertain which properties the excited scalar and pseudoscalar ${\bar{q}}q$ states possess and whether they can be identified in the physical spectrum.

Our study of the excited mesons is based on the Linear Sigma Model [77–80]. This is an effective approach to low-energy QCD – its degrees of freedom are not quarks and gluons of the underlying theory but rather meson fields with various values of *I*, *J*, *P* and *C*.

There are several advantages that the model has to offer. Firstly, it implements the symmetries of QCD as well as their breaking (see Sect. 2 for details). Secondly, it contains degrees of freedom with quantum numbers equal to those observed experimentally and in theoretical first-principles spectra (such as those of lattice QCD). This combination of symmetry-governed dynamics and states with correct quantum numbers justifies in our view the expectation that important aspects of the strong interaction are captured by the proposed model. Note that the model employed in this article is wide-ranging in that it contains the ground-state scalar, pseudoscalar, vector and axial-vector ${\bar{q}}q$ states in three flavours (*u*, *d*, *s*), the scalar dilaton (glueball) and the first excitations in the three-flavour scalar and pseudoscalar channels. Considering isospin multiplets as single degrees of freedom, there are 16 ${\bar{q}}q$ ground states and 8 ${\bar{q}}q$ excited states plus the scalar glueball in the model. For this reason, it can be denoted the “Extended Linear Sigma Model” (eLSM). A further advantage of eLSM is that the inclusion of degrees of freedom with a certain structure (such as ${\bar{q}}q$ states here) allows us to test the compatibility of experimentally known resonances with such structure. This is of immediate relevance for experimental hadron searches such as those planned at PANDA@FAIR [81].

With regard to vacuum states, the model has been used in studies of two-flavour ${\bar{q}}q$ mesons [82], glueballs [83–87], $K_1$ and other spin-1 mesons [88, 89] and baryons [90]. It is, however, also suitable for studies of the QCD phase diagram [91–93]. In this article, we will build upon the results obtained in Refs. [94, 95] where ground-state ${\bar{q}}q$ resonances and the glueball were considered in vacuum. Comparing experimental masses and decay widths with the theoretical predictions for excited states, we will draw conclusions on structure of the observed states; we will also predict more than 35 decays for various scalar and pseudoscalar resonances (see Sect. 3.3).

Irrespective of the above advantages, we must note that the model used in this article also has drawbacks. There are two that appear to be of particular importance.

Firstly, some of the states that might be of relevance in the region of interest are absent. The most important example is the scalar glueball whose mass is comparable [54, 58, 61, 64, 65] to that of the excited ${\bar{q}}q$ states discussed here. The implementation of the scalar glueball is actually straightforward in our approach (see Sect. 2) but the amount of its mixing with excited states is as yet unestablished, mainly due to the unfortunate lack of experimental data (discussed in Sect. 2.3.1).

Secondly, our calculations of decay widths are performed at tree level. Consequently, unitarity corrections are not included. A systematic way to implement them is to consider mesonic loops and determine their influence on the pole positions of resonances. Substantial shift of the pole position may then improve (or spoil) the comparison to the experimental data. However, the results of Ref. [96] suggest that unitarity corrections are small for resonances whose ratio of decay width to mass is small as well. Since such resonances are present in this article (see Sect. 3.3.3), the corrections will not be considered here.

Excited mesons were a subject of interest already several decades ago [97, 98]; to date, they have been considered in a wide range of approaches including QCD models/chiral Lagrangians [99–104], Lattice QCD [105–110], Bethe-Salpeter equation [111–114], NJL Model and its extensions [115–125], light-cone models [126], QCD string approaches [127] and QCD domain walls [128]. Chiral symmetry has also been suggested to become effectively restored in excited mesons [129, 130] rendering their understanding even more important. A study analogous to ours (including both scalar and pseudoscalar excitations and their various decay channels) was performed in extensions of the NJL model [117–119, 121, 122]. The conclusion was that $f_0(1370)$, $f_0(1710)$ and $a_0(1450)$ are the first radial excitations of $f_0(500)$, $f_0(980)$ and $a_0(980)$. However, this is at the expense of having very large decay widths for $f_0(1370)$, $f_0(1500)$ and $f_0(1710)$; in our case the decay widths for $f_0$ states above 1 GeV correspond to experimental data but the resonances are identified as quarkonium ground states [94].

The outline of the article is as follows. The general structure and results obtained so far regarding ground-state ${\bar{q}}q$ resonances are briefly reviewed in Sects. 2.1 and 2.2. Building upon that basis, we present the Lagrangian for the excited states and discuss the relevant experimental data in Sect. 2.3. Two hypotheses are tested in Sect. 3: whether the $f_0(1790)$ and $a_0(1950)$ resonances can represent excited ${\bar{q}}q$ states; the first one is not (yet) listed by the PDG but has been observed by the BES II and LHCb Collaborations [131, 132] and is discussed in Sect. 2.3.1. We also discuss to what extent it is possible to interpret the pseudoscalar mesons $\eta (1295)$, $\pi (1300)$, $\eta (1440)$ and *K*(1460) as excited states. Conclusions are presented in Sect. 4 and all interaction Lagrangians used in the model can be found in Appendix A. Our units are $\hbar = c = 1$; the metric tensor is $g_{\mu \nu } =$ diag$(+,-,-,-)$.

## The model

### General remarks

A viable effective approach to phenomena of non-perturbative strong interaction must implement the symmetries present in the underlying theory, QCD. The theory itself is rich in symmetries: colour symmetry $SU(3)_{c}$ (local); chiral $U(N_{f})_{L}\times U(N_{f})_{R}$ symmetry (*L* and *R* denote the ’left’ and ’right’ components and $N_{f}$ the number of quark flavours; global, broken in vacuum spontaneously by the non-vanishing chiral condensate $\langle {\bar{q}}q\rangle$ [133, 134], at the quantum level via the axial $U(1)_{A}$ anomaly [135] and explicitly by the non-vanishing quark masses); dilatation symmetry (broken at the quantum level [136, 137] but valid classically in QCD without quarks); *CPT* symmetry (discrete; valid individually for charge conjugation *C*, parity transformation *P* and time reversal *T*); $Z_{3}$ symmetry (discrete; pertaining to the centre elements of a special unitary matrix of dimension $N_{f}\times N_{f}$; non-trivial only at non-zero temperatures [138–143]) – all of course in addition to the Poincaré symmetry.Terms entering the Lagrangian of an effective approach to QCD should as a matter of principle be compatible with all symmetries listed above. Our subject is QCD in vacuum. In this context, we note that the colour symmetry is automatically fulfilled since we will be working with colour-neutral degrees of freedom; the structure and number of terms entering the Lagrangian are then restricted by the chiral, CPT and dilatation symmetries.

The eLSM Lagrangian has the following general structure:

1 $$\begin{aligned} \mathcal {L}=\mathcal {L}_{dil.}+\mathcal {L}_{0}+\mathcal {L}_{E} \end{aligned}$$

and in Sects. 2.2 and 2.3 we discuss the structure of the Lagrangians contributing to $\mathcal {L}$ as well as their matter content.

### Ground-state Quarkonia and Dilaton: Lagrangian and the matter content

This section contains a brief overview of the results obtained so far in the Extended Linear Sigma Model that contains $N_{f}=3$ scalar, pseudoscalar, vector and axial-vector quarkonia and the scalar glueball. The discussion is included for convenience of the reader and in order to set the basis for the incorporation of the excited quarkonia (Sect. 2.3). All details can be found in Refs. [94, 95].

In Eq. (1), $\mathcal {L}_{dil}$ implements, at the composite level, the dilatation symmetry of QCD and its breaking [144–149]:

2 $$\begin{aligned} \mathcal {L}_{dil.}=\frac{1}{2}(\partial _{\mu }G)^{2}-\frac{1}{4}\frac{m_{G} ^{2}}{\Lambda ^{2}}\left( G^{4}\ln \frac{G^{2}}{\Lambda ^{2}}-\frac{G^{4}}{4}\right) \end{aligned}$$

where *G* represents the dilaton field and $\Lambda$ is the scale that explicitly breaks the dilatation symmetry. Considering fluctuations around the potential minimum $G_{0}\equiv \Lambda$ leads to the emergence of a particle with $J^{PC}=0^{++}$ – the scalar glueball [83, 95].

Terms that (i) are compatible in their structure with the chiral, dilatation and CPT symmetries of QCD and (ii) contain ground-state scalar, pseudoscalar, vector and axial-vector quarkonia with $N_{f}=3$ and the dilaton are collected in the $\mathcal {L}_{0}$ contribution to Eq. (1), as in Refs. [82, 94, 95]:

3 $$\begin{aligned} \mathcal {L}_{0}&=\mathop {\mathrm {Tr}}[(D_{\mu }\Phi )^{\dagger }(D_{\mu }\Phi )]-m_{0}^{2}\left( \frac{G}{G_{0}}\right) ^{2}\mathop {\mathrm {Tr}}(\Phi ^{\dagger }\Phi )\nonumber \\&\quad -\,\lambda _{1}[\mathop {\mathrm {Tr}}(\Phi ^{\dagger }\Phi )]^{2}-\lambda _{2}\mathop {\mathrm {Tr}}(\Phi ^{\dagger }\Phi )^{2}\nonumber \\&\quad -\,\frac{1}{4}\mathop {\mathrm {Tr}}(L_{\mu \nu }^{2}+R_{\mu \nu }^{2} )\nonumber \\&\quad +\,\mathop {\mathrm {Tr}}\left[ \left( \left( \frac{G}{G_{0}}\right) ^{2}\frac{m_{1}^{2}}{2}+\Delta \right) \right. \nonumber \\&\left. \quad \times \, (L_{\mu }^{2}+R_{\mu }^{2})\right] +\mathop {\mathrm {Tr}}[H(\Phi +\Phi ^{\dagger })] \nonumber \\&\quad +\,\mathop {\mathrm {Tr}}(\Phi ^{\dagger }\Phi E_{0}+\Phi \Phi ^{\dagger }E_{0}) +c_{1}(\det \Phi -\det \Phi ^{\dagger })^{2}\nonumber \\&\quad +\,i\frac{g_{2}}{2} (\mathop {\mathrm {Tr}}\{L_{\mu \nu }[L^{\mu },L^{\nu } ]\}+\mathop {\mathrm {Tr}}\{R_{\mu \nu }[R^{\mu },R^{\nu }]\})\nonumber \\&\quad +\,\frac{h_{1}}{2}\mathop {\mathrm {Tr}}(\Phi ^{\dagger }\Phi )\mathop {\mathrm {Tr}}(L_{\mu }^{2}+R_{\mu }^{2})+h_{2} \mathop {\mathrm {Tr}}[\vert L_{\mu }\Phi \vert ^{2}\nonumber \\&\quad +\,\vert \Phi R_{\mu } \vert ^{2}]+2h_{3} \mathop {\mathrm {Tr}}(L_{\mu }\Phi R^{\mu }\Phi ^{\dagger })\nonumber \\&\quad +\,g_{3}[\mathop {\mathrm {Tr}}(L_{\mu }L_{\nu }L^{\mu }L^{\nu } )+\mathop {\mathrm {Tr}}(R_{\mu }R_{\nu }R^{\mu }R^{\nu })]\nonumber \\&\quad +\,g_{4} [\mathop {\mathrm {Tr}}\left( L_{\mu }L^{\mu }L_{\nu }L^{\nu }\right) +\mathop {\mathrm {Tr}}\left( R_{\mu }R^{\mu }R_{\nu }R^{\nu }\right) ]\nonumber \\&\quad +\,g_{5}\mathop {\mathrm {Tr}}\left( L_{\mu }L^{\mu }\right) \,\mathop {\mathrm {Tr}}\left( R_{\nu }R^{\nu }\right) {+}g_{6} [\mathop {\mathrm {Tr}}(L_{\mu }L^{\mu })\,\mathop {\mathrm {Tr}}(L_{\nu }L^{\nu })\nonumber \\&\quad +\,\mathop {\mathrm {Tr}}(R_{\mu }R^{\mu })\,\mathop {\mathrm {Tr}}(R_{\nu }R^{\nu })]. \end{aligned}$$

In Eq. (3), the matrices $\Phi$, $L^{\mu }$, and $R^{\mu }$ represent the scalar and vector nonets:

4 $$\begin{aligned}&\Phi =\sum _{i=0}^{8}(S_{i}+iP_{i})T_{i}=\frac{1}{\sqrt{2}}\nonumber \\&\quad \times \,\left( \begin{array} [c]{ccc} \frac{(\sigma _{N}+a_{0}^{0})+i(\eta _{N}+\pi ^{0})}{\sqrt{2}} &{} a_{0}^{+} +i\pi ^{+} &{} K_{0}^{\star +}+iK^{+}\\ a_{0}^{-}+i\pi ^{-} &{} \frac{(\sigma _{N}-a_{0}^{0})+i(\eta _{N}-\pi ^{0})}{\sqrt{2}} &{} K_{0}^{\star 0}+iK^{0}\\ K_{0}^{\star -}+iK^{-} &{} {{\bar{K}}_{0}^{\star 0}}+i{{\bar{K}}^{0}} &{} \sigma _{S}+i\eta _{S} \end{array} \right) ,\end{aligned}$$

5 $$\begin{aligned}&L^{\mu } =\sum _{i=0}^{8}(V_{i}^{\mu }+A_{i}^{\mu })T_{i}=\frac{1}{\sqrt{2} }\nonumber \\&\quad \times \,\left( \begin{array} [c]{ccc} \frac{\omega _{N}+\rho ^{0}}{\sqrt{2}}+\frac{f_{1N}+a_{1}^{0}}{\sqrt{2}} &{} \rho ^{+}+a_{1}^{+} &{} K^{\star +}+K_{1}^{+}\\ \rho ^{-}+a_{1}^{-} &{} \frac{\omega _{N}-\rho ^{0}}{\sqrt{2}}+\frac{f_{1N} -a_{1}^{0}}{\sqrt{2}} &{} K^{\star 0}+K_{1}^{0}\\ K^{\star -}+K_{1}^{-} &{} {{\bar{K}}}^{\star 0}+{{\bar{K}}}_{1}^{0} &{} \omega _{S}+f_{1S} \end{array} \right) ^{\mu },\end{aligned}$$

6 $$\begin{aligned}&R^{\mu } =\sum _{i=0}^{8}(V_{i}^{\mu }-A_{i}^{\mu })T_{i}=\frac{1}{\sqrt{2} }\nonumber \\&\quad \times \,\left( \begin{array} [c]{ccc} \frac{\omega _{N}+\rho ^{0}}{\sqrt{2}}-\frac{f_{1N}+a_{1}^{0}}{\sqrt{2}} &{} \rho ^{+}-a_{1}^{+} &{} K^{\star +}-K_{1}^{+}\\ \rho ^{-}-a_{1}^{-} &{} \frac{\omega _{N}-\rho ^{0}}{\sqrt{2}}-\frac{f_{1N} -a_{1}^{0}}{\sqrt{2}} &{} K^{\star 0}-K_{1}^{0}\\ K^{\star -}-K_{1}^{-} &{} {{\bar{K}}}^{\star 0}-{{\bar{K}}}_{1}^{0} &{} \omega _{S}-f_{1S} \end{array} \right) ^{\mu }, \end{aligned}$$

where $T_{i}\,(i=0,\ldots ,8)$ denote the generators of *U*(3), while $S_{i}$ represents the scalar, $P_{i}$ the pseudoscalar, $V_{i}^{\mu }$ the vector, $A_{i}^{\mu }$ the axial-vector meson fields. (Note that we are using the non-strange–strange basis defined as $\varphi _{N}=\frac{1}{\sqrt{3}}\left( \sqrt{2}\;\varphi _{0}+\varphi _{8}\right)$ and $\varphi _{S}=\frac{1}{\sqrt{3}}\left( \varphi _{0}-\sqrt{2}\;\varphi _{8}\right)$ with $\varphi \in (S_{i},P_{i} ,V_{i}^{\mu },A_{i}^{\mu })$.)

Furthermore,

7 $$\begin{aligned} D^{\mu }\Phi \equiv \partial ^{\mu }\Phi -ig_{1}(L^{\mu }\Phi -\Phi R^{\mu }) \end{aligned}$$

is the derivative of $\Phi$ transforming covariantly with regard to the $U(3)_{L}\times U(3)_{R}$ symmetry group; the left-handed and right-handed field strength tensors $L^{\mu \nu }$ and $R^{\mu \nu }$ are, respectively, defined as

8 $$\begin{aligned}&L^{\mu \nu } \equiv \partial ^{\mu }L^{\nu } - \partial ^{\nu }L^{\mu }, \end{aligned}$$

9 $$\begin{aligned}&R^{\mu \nu } \equiv \partial ^{\mu }R^{\nu } - \partial ^{\nu }R^{\mu }. \end{aligned}$$

The following symmetry-breaking mechanism is implemented:

- The spontaneous breaking of the $U(3)\times U(3)$ chiral symmetry requires setting $m_{0}^{2}<0$.
- The explicit breaking of the $U(3)\times U(3)$ chiral as well as dilatation symmetries is implemented by terms describing non-vanishing quark masses: $H=$ diag$\{h_{N},h_{N},h_{S}\}$, $\Delta =$ diag$\{0,0,\delta _{S}\}$ and $E_{0}=$ diag$\{0,0,\epsilon _{S}\}$.
- The $U(1)_{A}$ (chiral) anomaly is implemented by the determinant term $c_{1}(\det \Phi -\det \Phi ^{\dagger })^{2}$ [150, 151].

We also note the following important points:

- All states present in the Lagrangian (3), except for the dilaton, possess the ${\bar{q}}q$ structure [82, 152]. The argument is essentially based on the large-$N_{c}$ behaviour of the model parameters and on the model construction in terms of the underlying (constituent) quark fields. The ground-state Lagrangian (3) contains a pseudoscalar field assigned to the pion since it emerges from spontaneous breaking of the (chiral) $U(3) \times U(3)$ symmetry. Furthermore, the vector meson decaying into $2\pi$ is identified with the rho since the latter is experimentally known to decay into pions with a branching ratio of slightly less than 1. Pion and rho can be safely assumed to represent (very predominant) ${\bar{q}}q$ states and hence the large-$N_c$ behaviour of their mass terms has to be $N_c^0$. Additionally, the rho-pion vertex has to scale as $N_c^{-1/2}$ since the states are quarkonia. Then, as shown in Ref. [82], this is sufficient to determine the large-$N_c$ behaviour of all ground-state model parameters and of the non-strange and strange quark condensates. As a consequence, the masses of all other ground states scale as $N_c^0$ and their decay widths scale as $1/N_c$. For this reason, we identify these degrees of freedom with ${\bar{q}}q$ states. A further reason is that all states entering the matrix $\Phi$ in Eq. (4) can be decomposed in terms of (constituent) quark currents whose behaviour under chiral transformation is such that all terms in the Lagrangian (except for symmetry-breaking or anomalous ones) are chirally symmetric [152]. Note that our excited-state Lagrangian (16) will have exactly the same structure as the ground-state one. Considering the above discussion, we conclude that its degrees of freedom also have the ${\bar{q}}q$ structure.
- The number of terms entering Eq. (3) is finite under the requirements that (*i*) all terms are dilatationally invariant and hence have mass dimension equal to four, except possibly for those that are explicitly symmetry breaking or anomalous, and (*ii*) no term leads to singularities in the potential in the limit $G\rightarrow 0$ [153].
- Notwithstanding the above point, the glueball will not be a subject of this work – hence $G \equiv G_{0}$ is set throughout this article. With regard to the ground-state mesons, we will be relying on Ref. [94] since it contains the latest results from the model without the glueball. (For the model version with three-flavour ${\bar{q}}q$ states as well as the scalar glueball; see Ref. [95].)
- There are two scalar isospin-0 fields in the Lagrangian (3): $\sigma _{N}\equiv \bar{n}n$ (*n*: *u* and *d* quarks, assumed to be degenerate) and $\sigma _{S}\equiv \bar{s}s$. Spontaneous breaking of the chiral symmetry implies the existence of their respective vacuum expectation values $\phi _{N}$ and $\phi _{S}$. As described in Ref. [94], shifting of $\sigma _{N,S}$ by $\phi _{N,S}$ leads to the mixing of spin-1 and spin-0 fields. These mixing terms are removed by suitable shifts of the spin-1 fields that have the following general structure:
  10 $$\begin{aligned} V^{\mu }\rightarrow V^{\mu }+Z_{S}w_{V}\partial ^{\mu }S, \end{aligned}$$
  where $V^{\mu }$ and *S*, respectively, denote the spin-1 and spin-0 fields. The new constants $Z_{S}$ and $w_{V}$ are field-dependent and read [94]
  11 $$\begin{aligned}&w_{f_{1N}} =w_{a_{1}}=\frac{g_{1}\phi _{N}}{m_{a_{1}}^{2}}w_{f_{1S}}=\frac{\sqrt{2}g_{1}\phi _{S}}{m_{f_{1S}}^{2}}\nonumber \\&w_{K^{\star }}=\frac{ig_{1}(\phi _{N}-\sqrt{2}\phi _{S})}{2m_{K^{\star }}^{2} }w_{K_{1}}=\frac{g_{1}(\phi _{N}+\sqrt{2}\phi _{S})}{2m_{K_{1}}^{2} },\end{aligned}$$
  12 $$\begin{aligned}&Z_{\pi } =Z_{\eta _{N}}=\frac{m_{a_{1}}}{\sqrt{m_{a_{1}}^{2}-g_{1}^{2} \phi _{N}^{2}}}Z_{K}\nonumber \\&\quad =\frac{2m_{K_{1}}}{\sqrt{4m_{K_{1}}^{2} -g_{1}^{2}(\phi _{N}+\sqrt{2}\phi _{S})^{2}}},\end{aligned}$$
  13 $$\begin{aligned}&Z_{\eta _{S}} =\frac{m_{f_{1S}}}{\sqrt{m_{f_{1S}}^{2}-2g_{1}^{2}\phi _{S}^{2}}}Z_{K_{0}^{\star }} \nonumber \\&\quad =\frac{2m_{K^{\star }}}{\sqrt{4m_{K^{\star }}^{2}-g_{1}^{2}(\phi _{N}-\sqrt{2}\phi _{S})^{2}}}\text {.} \end{aligned}$$
  As demonstrated in Ref. [94], $\phi _{N}$ and $\phi _{S}$ are functions of $Z_{\pi }$ and $Z_{K}$ as follows:
  14 $$\begin{aligned} \phi _{N}&=Z_{\pi }f_{\pi }\end{aligned}$$
  15 $$\begin{aligned} \phi _{S}&=\sqrt{2}Z_{K}f_{K}-\phi _{N}/\sqrt{2}\; \end{aligned}$$
  where $f_{\pi }$ and $f_{K}$, respectively, denote the pion and kaon decay constants.

The ground-state mass terms can be obtained from Lagrangian (3); their explicit form can be found in Ref. [94] where a comprehensive fit of the experimentally known meson masses was performed. Fit results that will be used in this article are collected in Table 1. The following is of importance here:

- Table 1 contains no statement on masses and assignment of the isoscalar states $\sigma _{N}$ and $\sigma _{S}$. The reason is that their identification in the meson spectrum is unclear due to both theoretical and experimental uncertainties [154, 155]. In Ref. [94], the preferred assignment of $\sigma _{N}$ was to $f_{0}(1370)$, not least due to the best-fit result $m_{\sigma _{N}} = 1363$ MeV. The resonance $\sigma _{S}$ was assigned to $f_{0}(1710)$. Note that a subsequent analysis in Ref. [95], which included the scalar glueball, found the assignment of $\sigma _{S}$ to $f_{0}(1500)$ more preferable; $f_0(1710)$ was found to be compatible with the glueball. These issues will be of secondary importance here since no mixing between excited and ground states will be considered. (We also note that decays of the excited states into $f_0(1500)$ and $f_0(1710)$ would be kinematically forbidden. Excited-state masses are discussed in Sect. 3).
- Table 1 also contains no statement on the axial-vector kaon $K_{1}$. Reference [94] obtained $m_{K_{1}} = 1282$ MeV as the best-fit result. One needs to note, however, that PDG listings [5] contain two states to which our $K_{1}$ resonance could be assigned: $K_{1}(1270)$ and $K_{1}(1400)$. Both have a significant mutual overlap [156–159, 161, 161–174]; analysis from the Linear Sigma Model suggests that our $K_{1}$ state has a larger overlap with $K_{1}(1400)$ [89]. Nonetheless, we will use $m_{K_{1}} = 1282$ MeV for decays of excited states involving $K_{1}$ – this makes no significant difference to our results since the decays with $K_{1}$ final states are phase-space suppressed for the mass range of excited mesons.
- The states $\eta$ and $\eta ^{\prime }$ arise from mixing of $\eta _{N}$ and $\eta _{S}$ in Lagrangian (3). The mixing angle is $\theta _{\eta }=-44.6^{\circ }$ [94]; see also Refs. [175–183].

**Table 1:** Best-fit results for masses of ground-state mesons and pseudoscalar decay constants present in Eq. (3), obtained in Ref. [94]. The values in the third column will be used in this article in order for us to remain model-consistent. Note that the errors in the fourth column correspond either to the experimental values or to 5% of the respective central values (whichever is larger)

Observable	Model ground state assigned to	Fit (MeV)	Experiment (MeV)
$m_{\pi }$	Pion	$141.0 \pm 5.8$	$137.3 \pm 6.9$
$m_{K}$	Kaon	$485.6 \pm 3.0$	$495.6 \pm 24.8$
$m_{\eta }$	$\eta$	$509.4 \pm 3.0$	$547.9 \pm 27.4$
$m_{\eta ^{\prime }}$	$\eta ^{\prime }(958)$	$962.5 \pm 5.6$	$957.8 \pm 47.9$
$m_{\rho } \equiv m_{\omega _{N}}$	$\rho (770)$	$783.1 \pm 7.0$	$775.5 \pm 38.8$
$m_{K^{\star }}$	$K^{\star }(892)$	$885.1 \pm 6.3$	$893.8 \pm 44.7$
$m_{\phi }$	$\phi (1020)$	$975.1 \pm 6.4$	$1019.5 \pm 51.0$
$m_{a_{1}} \equiv m_{f_{1N}}$	$a_{1}(1260)$	$1186 \pm 6$	$1230 \pm 62$
$m_{f_{1S}}$	$f_{1}(1420)$	$1372.5 \pm 5.3$	$1426.4 \pm 71.3$
$m_{a_{0}}$	$a_{0}(1450)$	$1363 \pm 1$	$1474 \pm 74$
$m_{K_{0}^{\star }}$	$K_{0}^{\star }(1430)$	$1450 \pm 1$	$1425 \pm 71$
$f_{\pi }$	–	$96.3 \pm 0.7$	$92.2 \pm 4.6$
$f_{K}$	–	$106.9 \pm 0.6$	$110.4 \pm 5.5$

### Excited scalars and pseudoscalars

#### Lagrangian

With the foundations laid in the previous section, the most general Lagrangian for the excited scalar and pseudoscalar quarkonia with terms up to order four in the naive scaling can be constructed as follows:

16 $$\begin{aligned} \mathcal {L}_{E}&=\mathop {\mathrm {Tr}}[(D_{\mu }\Phi _E)^{\dagger }(D_{\mu }\Phi _E)]+\alpha \mathop {\mathrm {Tr}}[(D_{\mu }\Phi _E)^{\dagger }(D_{\mu }\Phi )\nonumber \\&\quad +\, (D_{\mu }\Phi )^{\dagger }(D_{\mu }\Phi _E)]-(m_{0} ^{*})^{2}\left( \frac{G}{G_{0}}\right) ^{2}\mathop {\mathrm {Tr}}(\Phi _{E}^{\dagger }\Phi _{E})\nonumber \\&\quad -\,\lambda _{0}\left( \frac{G}{G_{0}}\right) ^{2}\mathop {\mathrm {Tr}}(\Phi _{E}^{\dagger }\Phi +\Phi ^{\dagger }\Phi _{E})\nonumber \\&\quad -\lambda _{1}^{*} \mathop {\mathrm {Tr}}(\Phi _{E}^{\dagger }\Phi _{E})\mathop {\mathrm {Tr}}(\Phi ^{\dagger }\Phi )\nonumber \\&\quad -\,\lambda _{2}^{*}\mathop {\mathrm {Tr}}(\Phi _{E}^{\dagger } \Phi _{E}\Phi ^{\dagger }\Phi +\Phi _{E}\Phi _{E}^{\dagger }\Phi \Phi ^{\dagger })\nonumber \\&\quad -\,\kappa _{1}\mathop {\mathrm {Tr}}(\Phi _{E}^{\dagger }\Phi +\Phi ^{\dagger } \Phi _{E})\mathop {\mathrm {Tr}}(\Phi ^{\dagger }\Phi )\nonumber \\&\quad -\kappa _{2} [\mathop {\mathrm {Tr}}(\Phi _{E}^{\dagger }\Phi +\Phi ^{\dagger }\Phi _{E} )]^{2}\nonumber \\&\quad -\,\kappa _{3}\mathop {\mathrm {Tr}}(\Phi _{E}^{\dagger }\Phi +\Phi ^{\dagger }\Phi _{E})\mathop {\mathrm {Tr}}(\Phi _{E}^{\dagger }\Phi _{E}) \nonumber \\&\quad -\,\kappa _{4}[\mathop {\mathrm {Tr}}(\Phi _{E}^{\dagger }\Phi _{E})]^{2}\nonumber \\&\quad -\,\xi _{1}\mathop {\mathrm {Tr}}(\Phi _{E}^{\dagger }\Phi \Phi ^{\dagger }\Phi +\Phi _{E}\Phi ^{\dagger }\Phi \Phi ^{\dagger })\nonumber \\&\quad -\,\xi _{2}\mathop {\mathrm {Tr}}(\Phi _{E}^{\dagger }\Phi \Phi _{E}^{\dagger }\Phi +\Phi ^{\dagger }\Phi _{E}\Phi ^{\dagger }\Phi _{E})\nonumber \\&\quad -\,\xi _{3}\mathop {\mathrm {Tr}}(\Phi ^{\dagger }\Phi _{E}\Phi _{E} ^{\dagger }\Phi _{E}+\Phi \Phi _{E}^{\dagger }\Phi _{E}\Phi _{E}^{\dagger })\nonumber \\&\quad -\xi _{4}\mathop {\mathrm {Tr}}(\Phi _{E}^{\dagger }\Phi _{E})^{2}\nonumber \\&\quad +\,\mathop {\mathrm {Tr}}(\Phi _{E}^{\dagger }\Phi _{E}E_{1}+\Phi _{E}\Phi _{E}^{\dagger }E_{1})\nonumber \\&\quad +c_{1}^{*} [(\det \Phi -\det \Phi _{E}^{\dagger })^{2}\nonumber \\&\quad +\,(\det \Phi ^{\dagger }-\det \Phi _{E})^{2}]+c_{1E}^{*}(\det \Phi _{E}-\det \Phi _{E}^{\dagger })^{2}\nonumber \\&\quad +\,\frac{h_{1}^{*}}{2}\mathop {\mathrm {Tr}}(\Phi _{E}^{\dagger }\Phi +\Phi ^{\dagger }\Phi _{E})\mathop {\mathrm {Tr}}(L_{\mu }^{2}+R_{\mu }^{2} )\nonumber \\&\quad +\,\frac{h_{1E}^{*}}{2}\mathop {\mathrm {Tr}}(\Phi _{E}^{\dagger }\Phi _{E})\mathop {\mathrm {Tr}}(L_{\mu }^{2}+R_{\mu }^{2})\nonumber \\&\quad +\,h_{2}^{*}\mathop {\mathrm {Tr}}(\Phi _{E}^{\dagger }L_{\mu }L^{\mu }\Phi +\Phi ^{\dagger }L_{\mu }L^{\mu }\Phi _{E}\nonumber \\&\quad +\,R_{\mu }\Phi _{E}^{\dagger }\Phi R^{\mu }+R_{\mu }\Phi ^{\dagger }\Phi _{E}R^{\mu })\nonumber \\&\quad +h_{2E}^{*} \mathop {\mathrm {Tr}} [\vert L_{\mu }\Phi _E \vert ^{2}+\vert \Phi _E R_{\mu } \vert ^{2}]\nonumber \\&\quad +\,2h_{3}^{*}\mathop {\mathrm {Tr}}(L_{\mu }\Phi _{E}R^{\mu }\Phi ^{\dagger }+L_{\mu }\Phi R^{\mu }\Phi _{E}^{\dagger })\nonumber \\&\quad +\,2h_{3E}^{*} \mathop {\mathrm {Tr}}(L_{\mu }\Phi _{E}R^{\mu }\Phi _{E}^{\dagger }). \end{aligned}$$

The particle content of the Lagrangian is the same as the one in Eqs. (5) and (6) for spin-1 states and it is analogous to Eq. (4) for (pseudo)scalars:

17 $$\begin{aligned}&\Phi _{E}=\frac{1}{\sqrt{2}}\nonumber \\&\quad \times \,\left( \begin{array}{lll} \frac{(\sigma _{N}^{E}+a_{0}^{0E})+i(\eta _{N}^{E}+\pi ^{0E})}{\sqrt{2}} &{} a_{0}^{+E}+i\pi ^{+E} &{} K_{0}^{\star +E}+iK^{+E}\\ a_{0}^{-E}+i\pi ^{-E} &{} \frac{(\sigma _{N}^{E}-a_{0}^{0E})+i(\eta _{N}^{E} -\pi ^{0E})}{\sqrt{2}} &{} K_{0}^{\star 0E}+iK^{0E}\\ K_{0}^{\star -E}+iK^{-E} &{} {{\bar{K}}_{0}^{\star 0E}}+i{{\bar{K}}^{0E}} &{} \sigma _{S}^{E}+i\eta _{S}^{E} \end{array} \right) .\nonumber \\ \end{aligned}$$

The covariant derivative $D^{\mu }\Phi ^{E}$ is defined analogously to Eq. (7):

18 $$\begin{aligned} D^{\mu }\Phi ^{E}\equiv \partial ^{\mu }\Phi ^{E}-ig_{1}^{E}(L^{\mu }\Phi ^{E} -\Phi ^{E}R^{\mu }) \end{aligned}$$

and we also set $E_{1}=$ diag$\{0,0,\epsilon _{S}^{E}\}$.

Spontaneous symmetry breaking in the Lagrangian for the excited (pseudo)scalars will be implemented only by means of condensation of ground-state quarkonia $\sigma _{N}$ and $\sigma _{S}$, i.e., as a first approximation, we assume that their excited counterparts $\sigma _{N}^{E}$ and $\sigma _{S}^{E}$ do not condense.^1^ As a consequence, there is no need to shift spin-1 fields or renormalise the excited pseudoscalars as described in Eqs. (10)–(11).

We now turn to the assignment of the excited states. Considering isospin multiplets as single degrees of freedom, there are 8 states in Eq. (17): $\sigma _{N}^{E}$, $\sigma _{S}^{E}$, $\mathbf {a}_{0}^{E}$ and $K_{0}^{\star E}$ (scalar) and $\eta _{N}^{E}$, $\eta _{S}^{E}$, ${\pi }^{E}$ and $K^{E}$ (pseudoscalar); the experimental information on states with these quantum numbers is at times limited or their identification is unclear:

- Seven states are listed by the PDG in the scalar isosinglet ($IJ^{PC} =00^{++}$) channel in the energy region up to $\simeq$ 2 GeV: $f_{0}(500)$/$\sigma$, $f_{0}(980)$, $f_{0}(1370)$, $f_{0}(1500)$, $f_{0}(1710)$, $f_{0}(2020)$ and $f_0(2100)$. The last two are termed unestablished [5]; the others have been subject of various studies in the last decades [10, 11, 15–52, 82, 94]. As mentioned in the Introduction, the general conclusion is that the states up to and including $f_{0}(1710)$ are compatible with having ground-state ${\bar{q}}q$ or ${\bar{q}}{\bar{q}}qq$ structure; the presence of the scalar glueball is also expected [42, 53–72, 83, 95]. However, none of these states is considered as the first radial excitation of the scalar isosinglet ${\bar{q}}q$ state.A decade ago, a new resonance named $f_{0}(1790)$ was observed by the BES II Collaboration in the $\pi \pi$ final states produced in $J/\Psi$ radiative decays [131]; there had been evidence for this state in the earlier data of MARK III [185] and BES [186]. Recently, LHCb has confirmed this finding in a study of $B_{s}\rightarrow J/\Psi \pi \pi$ decays [132]. Since, as indicated, the spectrum of ground-state scalar quarkonia appears to be contained in the already established resonances, we will work here with the hypothesis that $f_{0}(1790)$ is the first excitation of the $\bar{n}n$ ground state ($\equiv \sigma _{N}^{E}$). The assignment is further motivated by the predominant coupling of $f_0(1790)$ to pions [131]. The data of Ref. [131] will be used as follows: $m_{f_{0} (1790)}=(1790\pm 35)$ MeV and $\Gamma _{f_{0}(1790)\rightarrow \pi \pi } =(270\pm 45)$ MeV, with both errors made symmetric and given as arithmetic means of those published by BES II. Additionally, Ref. [131] also reports the branching ratios $J/\Psi \rightarrow \phi f_{0}(1790)\rightarrow \phi \pi \pi =(6.2\pm 1.4)\cdot 10^{-4}$ and $J/\Psi \rightarrow \phi f_{0}(1790)\rightarrow \phi KK=(1.6\pm 0.8)\cdot 10^{-4}$. Using $\Gamma _{f_{0}(1790)\rightarrow \pi \pi }=(270\pm 45)$ MeV and the quotient of the mentioned branching ratios we estimate $\Gamma _{f_{0}(1790)\rightarrow KK}=(70\pm 40)$ MeV. These data will become necessary in Sects. 3.2 and 3.3. We note, however, already at this point that the large uncertainties in $f_0(1790)$ decays – a direct consequence of uncertainties in the $J/\Psi$ branching ratios amounting to $\sim$23% and 50% – will lead to ambiguities in prediction of some decays (see Sect. 3.3.1). These are nonetheless the most comprehensive data available at the moment, and more data would obviously be of great importance. The assignment of our excited isoscalar $\bar{s}s$ state $\sigma _{S}^{E}$ will be discussed as a consequence of the model [particularly in the context of $f_{0}(2020)$ and $f_0(2100)$].
- Two resonances are denoted as established by the PDG in the $IJ^{PC}=10^{++}$ channel: $a_{0}(980)$ and $a_{0}(1450)$ [5]. Various interpretations of these two states in terms of ground-state ${\bar{q}}q$ or ${\bar{q}}{\bar{q}}qq$ structures or meson–meson molecules have been proposed [20, 23, 24, 26, 28, 30–32, 36–41, 43, 49, 52, 73, 74, 76].Recently, the BABAR Collaboration [187] has claimed the observation of a new resonance denoted $a_{0}(1950)$ in the process $\gamma \gamma \rightarrow \eta _{c}(1S)\rightarrow {\bar{K}}K\pi$ with significance up to 4.2 $\sigma$. There was earlier evidence for this state in the Crystal Barrel data [188, 189]; see also Refs. [190, 191]. We will discuss the possible interpretation of this resonance in terms of the first $IJ^{PC}=10^{++}$excitation as a result of our calculations.
- Two resonances are candidates for the ground-state ${\bar{q}}q$ resonance in the scalar-kaon channel (with alternative interpretations – just as in the case of the $a_{0}$ resonances – in terms of ${\bar{q}}{\bar{q}}qq$ structures or meson–meson molecules): $K_{0}^{\star }(800)$/$\kappa$ and $K_{0}^{\star }(1430)$; controversy still surrounds the first of these states [11, 20, 26, 28, 30–32, 34, 35, 37, 39, 49, 74–76]. A possibility is that $K_{0}^{\star }(1950)$, the highest-lying resonance in this channel, represents the first excitation, although the state is (currently) unestablished [5]. This will be discussed as a result of our calculations later on.
- The pseudoscalar isosinglet ($IJ^{PC}=00^{-+}$) channel has six known resonances in the energy region below 2 GeV according to the PDG [5]: $\eta$, $\eta ^{\prime }(958)$, $\eta (1295)$, $\eta (1405)$, $\eta (1475)$ and $\eta (1760)$. Not all of them are without controversy: for example, the observation of $\eta (1405)$ and $\eta (1475)$ as two different states was reported by E769 [192], E852 [193], MARK III [194], DM2 [195] and OBELIX [196, 197], while they were claimed to represent a single state named $\eta (1440)$ by the Crystal Ball [198] and BES [199, 200] Collaborations. It is important to note that a clear identification of pseudoscalar resonance(s) in the energy region between 1.4 GeV and 1.5 GeV depends strongly on a proper consideration, among other, of the $K^{\star }K$ threshold opening ($m_{K^{\star }}+m_{K}=1385$ MeV) and of the existence of the $IJ^{PC}=01^{++}$ state $f_{1}(1420)$ whose partial wave is known to influence the pseudoscalar one in experimental analyses (see, e.g., Ref. [193]). A comprehensive study of BES II data in Ref. [201], which included an energy-dependent Breit–Wigner amplitude as well as a dispersive correction to the Breit–Wigner denominator (made necessary by the proximity to the $K^{\star }K$ threshold), has observed only a marginal increase in fit quality when two pseudoscalars are considered. In line with this, our study will assume the existence of $\eta (1440)$ to which our $\eta _{S}^{E}$ state will be assigned. We will use $m_{\eta (1440)}=(1432\pm 10)$ MeV and $\Gamma _{\eta (1440) \rightarrow K^{\star } K} = (26 \pm 3)$ MeV [199, 200] in Sects. 3.2 and 3.3.2; the error in the decay width is our estimate. We emphasise, however, that our results are stable up to a $\lesssim$3% change when $\eta (1475)$ is considered instead of $\eta (1440)$.^2^

**Table 2:** Assignment of the states in Eq. (17) to physical states. Every assignment implies the hypothesis that the physical state has the ${\bar{q}}q$ structure

Model state	$IJ^{P}$	Assignment	We use
$\sigma _{N}^{E}$	$00^+$	$f_{0}(1790)$	$m_{f_0(1790)} = (1790 \pm 35)$ MeV [131] $\Gamma _{f_{0}(1790)\rightarrow \pi \pi }=(270\pm 45)$ MeV [131] $\Gamma _{f_{0}(1790)\rightarrow KK}=(70\pm 40)$ MeV
$\eta _{N}^{E}$	$00^-$	$\eta (1295)$	$m_{\eta (1295)}=(1294\pm 4)$ MeV [5] $\Gamma _{\eta (1295)}^{\text {total}} = (55 \pm 5)$ MeV [5]
$\eta _{S}^{E}$	$00^-$	$\eta (1440)$	$m_{\eta (1440)}=(1432\pm 10)$ MeV [199, 200] $\Gamma _{\eta (1440) \rightarrow K^{\star } K} = (26 \pm 3)$ MeV
$\sigma _{S}^{E}$	$00^+$	Possible overlap with $f_{0}(2020)$/$f_0(2100)$ to be discussed as a model consequence	–
$a_{0}^{E}$	$10^+$	Possible overlap with $a_{0}(1950)$ to be discussed as a model consequence	–
$\pi ^{E}$	$10^-$	Possible overlap with $\pi (1300)$ to be discussed as a model consequence	–
$K_{0}^{\star E}$	$\frac{1}{2} 0^+$	Possible overlap with $K_{0}^{\star }(1950)$ to be discussed as a model consequence	–
$K^{E}$	$\frac{1}{2} 0^-$	Possible overlap with *K*(1460) to be discussed as a model consequence	–

Our state $\eta _{N}^{E}$ will be assigned to $\eta (1295)$ in order to test the hypothesis whether an excited pseudoscalar isosinglet at $\simeq 1.3$ GeV can be accommodated in eLSM (and notwithstanding the experimental concerns raised in Ref. [203]). We will use the PDG value $m_{\eta (1295)}=(1294\pm 4)$ MeV for determination of mass parameters in Sect. 3.2. The PDG also reports $\Gamma _{\eta (1295)}^{\text {total}} = (55 \pm 5)$ MeV; the relative contributions of $\eta (1295)$ decay channels are uncertain. Nonetheless, we will use $\Gamma _{\eta (1295)}^{\text {total}}$ in Sect. 3.3.2.
- Two states have the quantum number of a pion excitation: $\pi (1300)$ and $\pi (1800)$, with the latter being a candidate for a non-${\bar{q}}q$ state [5]. The remaining $\pi (1300)$ resonance may in principle be an excited ${\bar{q}}q$ isotriplet; however, due to the experimental uncertainties reported by the PDG [$m_{\pi (1300)}=(1300\pm 100)$ MeV but merely an interval for $\Gamma _{\pi (1300)}=(200-600)$ MeV] this will only be discussed as a possible result of our model.
- Two states are candidates for the excited kaon: *K*(1460) and *K*(1830). Since other excited states of our model have been assigned to resonances with energies $\simeq$1.4 GeV, we will study the possibility that our $IJ^{P}=\frac{1}{2}0^{-}$ state corresponds to *K*(1460). This will, however, only be discussed as a possible result of the model since the experimental data on this state is very limited: $m_{K(1460)}\sim 1460$ MeV; $\Gamma _{K(1460)}\sim 260$ MeV [5].

As indicated in the above points, with regard to the use of the above data for parameter determination we exclude as input all states for which there are only scarce/unestablished data and, additionally, those for which the PDG cites only intervals for mass/decay width (since the latter lead to weak parameter constraints). Then we are left with only three resonances whose experimental data shall be used: $f_0(1790)$, $\eta (1295)$ and $\eta (1440)$. For clarity, we collect the assignment of the model states (where possible), and also the data that we will use, in Table 2. The data are used in Sect. 3.

#### Parameters

The following parameters are present in Eq. (16):

19 $$\begin{aligned}&g_{1}^{E},\alpha ,m_{0}^{*},\lambda _{0},\lambda _{1,2}^{*},\kappa _{1,2,3,4},\nonumber \\&\xi _{1,2,3,4},\epsilon _{S}^{E},c_{1}^{*},c_{1}^{*E},h_{1,2,3}^{*},h_{1,2,3}^{*E}. \end{aligned}$$

The number of parameters relevant for masses and decays of the excited states is significantly smaller as apparent once the following selection criteria are applied:

- All large-$N_{c}$ suppressed parameters are set to zero since their influence on the general phenomenology is expected to be small and the current experimental uncertainties do not permit their determination. Hence the parameters $\lambda _{1}^{*}$, $h_{1}^{*}$ and $\kappa _{1,2,3,4}$ are discarded.
- The parameter $c_{1}^{*}$ is set to zero since it contains a term $\sim (\det \Phi )^{2}$, which would influence ground-state mass terms after condensation of $\sigma _{N}$ and $\sigma _{S}$. Such introduction of an additional parameter is not necessary since, as demonstrated in Ref. [94], the ground states are very well described by Lagrangian (3).
- As a first approximation, we will discard all parameters that lead to particle mixing and study whether the assignments described in Table 2 are compatible with experiment. Hence we discard the parameters $\alpha$, $\lambda _{0}$ and $\xi _{1}$; note that mixing is also induced by $\kappa _{1,2}$ and $c_{1}^{*}$ but these have already been discarded for reasons stated above.^3^
- Parameters that lead to decays with two or more excited final states are not of relevance for us: all states in the model have masses between $\sim 1$ GeV and $\sim 2$ GeV and hence such decays are kinematically forbidden. (Parameters $\lambda _{2}^{*}$ and $\xi _{2}$ that contribute to mass terms are obviously relevant and excepted from this criterion.) Hence we can discard $\xi _{3,4}$, $c_{1}^{*E}$ and $h_{1,2,3}^{*E}$.

Note that the above criteria are not mutually exclusive: some parameters may be set to zero on several grounds, such as for example $\kappa _{1}$.

Consequently we are left with the following undetermined parameters:

20 $$\begin{aligned} g_{1}^{E},m_{0}^{*},\lambda _{2}^{*},\xi _{2},\epsilon _{S}^{E},h_{2,3}^{*}. \end{aligned}$$

The number of parameters that we will actually use is even smaller, as we discuss in Sects. 2.3.3 and 2.3.4.

#### Mass terms

The following mass terms are obtained for the excited states present in the model:

21 $$\begin{aligned}&m_{\sigma _{N}^{E}}^{2} =(m_{0}^{*})^{2}+\frac{\lambda _{2}^{*} +\xi _{2}}{2}\phi _{N}^{2},\end{aligned}$$

22 $$\begin{aligned}&m_{a_{0}^{E}}^{2} =(m_{0}^{*})^{2}+\frac{\lambda _{2}^{*}+\xi _{2}}{2}\phi _{N}^{2},\end{aligned}$$

23 $$\begin{aligned}&m_{\pi ^{E}}^{2} =m_{\eta _{N}^{E}}^{2}=(m_{0}^{*})^{2}+\frac{\lambda _{2}^{*}-\xi _{2}}{2}\phi _{N}^{2},\end{aligned}$$

24 $$\begin{aligned}&m_{\eta _{S}^{E}}^{2} =(m_{0}^{*})^{2}-2\epsilon _{S}^{E}+\left( \lambda _{2}^{*}-\xi _{2}\right) \phi _{S}^{2}\end{aligned}$$

25 $$\begin{aligned}&m_{\sigma _{S}^{E}}^{2} =(m_{0}^{*})^{2}-2\epsilon _{S}^{E}+\left( \lambda _{2}^{*}+\xi _{2}\right) \phi _{S}^{2},\end{aligned}$$

26 $$\begin{aligned}&m_{K^{E}}^{2} =(m_{0}^{*})^{2}-\epsilon _{S}^{E}+\frac{\lambda _{2} ^{*}}{4}\phi _{N}^{2}\nonumber \\&\qquad \qquad -\,\frac{\xi _{2}}{\sqrt{2}}\phi _{N}\phi _{S}+\frac{\lambda _{2}^{*}}{2}\phi _{S}^{2},\end{aligned}$$

27 $$\begin{aligned}&m_{K_{0}^{\star E}}^{2} =(m_{0}^{*})^{2}-\epsilon _{S}^{E}+\frac{\lambda _{2}^{*}}{4}\phi _{N}^{2}\nonumber \\&\qquad \qquad +\,\frac{\xi _{2}}{\sqrt{2}}\phi _{N}\phi _{S}+\frac{\lambda _{2}^{*}}{2}\phi _{S}^{2}. \end{aligned}$$

The mass terms (21)–(27) contain the same linear combination of $m_{0}^{*}$ and $\lambda _{2}^{*}$:

28 $$\begin{aligned} C_{1}^{*}=(m_{0}^{*})^{2}+\frac{\lambda _{2}^{*}}{2}\phi _{N}^{2}, \end{aligned}$$

and the mass terms (24)–(27) contain the same linear combination of $\lambda _{2}^{*}$ and $\epsilon _{S}^{E}$:

29 $$\begin{aligned} C_{2}^{*}=\lambda _{2}^{*}Z_{K}f_{K}(Z_{K}f_{K}-\phi _{N})-\epsilon _{S}^{E}. \end{aligned}$$

This is obvious after substituting the strange condensate $\phi _{S}$ by the non-strange condensate $\phi _{N}$ via Eq. (15). The modified mass terms then read

30 $$\begin{aligned}&m_{\sigma _{N}^{E}}^{2} =C_{1}^{*}+\frac{\xi _{2}}{2}\phi _{N} ^{2},\end{aligned}$$

31 $$\begin{aligned}&m_{a_{0}^{E}}^{2} =C_{1}^{*}+\frac{\xi _{2}}{2}\phi _{N}^{2} ,\end{aligned}$$

32 $$\begin{aligned}&m_{\pi ^{E}}^{2} =m_{\eta _{N}^{E}}^{2}=C_{1}^{*}-\frac{\xi _{2}}{2} \phi _{N}^{2} ,\end{aligned}$$

33 $$\begin{aligned}&m_{\eta _{S}^{E}}^{2} =C_{1}^{*}+2C_{2}^{*}-\frac{\xi _{2}}{2} (\phi _{N}-2Z_{K}f_{K})^{2} ,\end{aligned}$$

34 $$\begin{aligned}&m_{\sigma _{S}^{E}}^{2} =C_{1}^{*}+2C_{2}^{*}+\frac{\xi _{2}}{2} (\phi _{N}-2Z_{K}f_{K})^{2} ,\end{aligned}$$

35 $$\begin{aligned}&m_{K^{E}}^{2} =C_{1}^{*}+C_{2}^{*}+\frac{\xi _{2}}{2}\phi _{N} (\phi _{N}-2Z_{K}f_{K}) ,\end{aligned}$$

36 $$\begin{aligned}&m_{K_{0}^{\star \,E}}^{2} =C_{1}^{*}+C_{2}^{*}-\frac{\xi _{2}}{2} \phi _{N}(\phi _{N}-2Z_{K}f_{K}). \end{aligned}$$

Mass terms for all eight excited states can hence be described in terms of only three parameters from Eq. (16): $C_{1}^{*}$, $C_{2}^{*}$ and $\xi _{2}$.

#### Decay widths

Our objective is to perform a tree-level calculation of all kinematically allowed two- and three-body decays for all excited states present in the model. The corresponding interaction Lagrangians are presented in Appendix A. As we will see, there are more than 35 decays that can be determined in this way but all of them can be calculated using only a few formulae.

The generic formula for the decay width of particle *A* into particles *B* and *C* reads

37 $$\begin{aligned} \Gamma _{A\rightarrow BC}=\mathcal {I}\frac{|{k}|}{8\pi m_{A}^{2} }\left| \mathcal {M}_{A\rightarrow BC}\right| ^{2}, \end{aligned}$$

where *k* is the three-momentum of one of the final states in the rest frame of *A* and $\mathcal {M}$ is the decay amplitude (i.e., a transition matrix element). $\mathcal {I}$ is a symmetry factor emerging from the isospin symmetry – it is determined by the number of sub-channels for a given set of final states (e.g., $\mathcal {I}$ $=2$ if *B* and *C* both correspond to kaons). Usual symmetry factors are included if the final states are identical. As we will see in Sect. 3.3, decay widths obtained in the model are generally much smaller than resonance masses; for this reason, we do not expect large unitarisation effects [96].

Depending on the final states, the interaction Lagrangians presented in Appendix A can have one of the following general structures:

- For a decay of the form $S\rightarrow P_{1}P_{2}$, where *S* is a scalar and $P_{1}$ and $P_{2}$ are pseudoscalar particles, the generic structure of the interaction Lagrangian is
  38 $$\begin{aligned} \mathcal {L}_{SP_{1}P_{2}}= & {} D_{SP_{1}P_{2}}\,SP_{1}P_{2}+E_{SP_{1}P_{2} }\,S\partial _{\mu }P_{1}\partial ^{\mu }P_{2}\nonumber \\&+\,F_{SP_{1}P_{2}}\,\partial _{\mu }S\partial ^{\mu }P_{1}P_{2}, \end{aligned}$$
  where $D_{SP_{1}P_{2}}$, $E_{SP_{1}P_{2}}$ and $F_{SP_{1}P_{2}}$ are combinations of (some of the) parameters entering Lagrangian (16). According to Eq. (37), the decay width reads in this case
  39 $$\begin{aligned} \Gamma _{S\rightarrow P_{1}P_{2}}= & {} \mathcal {I}\frac{|{k}|}{8\pi m_{S} ^{2}}\vert D_{SP_{1}P_{2}}-E_{SP_{1}P_{2}}\,K_{1}\cdot K_{2}\nonumber \\&+\,F_{SP_{1}P_{2}}\,K\cdot K_{1}\vert ^{2}, \end{aligned}$$
  where *K*, $K_{1}$ and $K_{2}$ are respectively 4-momenta of *S*, $P_{1}$ and $P_{2}$.
- For a decay of the form $S\rightarrow VP$, where *V* is a vector and *P* is a pseudoscalar particle, the generic structure of the interaction Lagrangian is
  40 $$\begin{aligned} \mathcal {L}_{SVP}=D_{SVP}\,SV_{\mu }\partial ^{\mu }P, \end{aligned}$$
  where $D_{SVP}$ is a combination of (some of the) parameters entering Lagrangian (16). The decay width reads in this case
  41 $$\begin{aligned}&\Gamma _{S\rightarrow VP}=\mathcal {I}\frac{|{k}|}{8\pi m_{S}^{2}} D_{SVP}^{2}\nonumber \\&\times \,\left[ \frac{(m_{S}^{2}-m_{V}^{2}-m_{P}^{2})^{2}}{4m_{V}^{2} }-m_{P}^{2}\right] . \end{aligned}$$
- For a decay of the form $S\rightarrow V_{1}V_{2}$, where $V_{1}$ and $V_{2}$ are vector particles, the generic structure of the interaction Lagrangian is
  42 $$\begin{aligned} \mathcal {L}_{SV_{1}V_{2}}=D_{SV_{1}V_{2}}\,SV_{1\mu }V_{2}^{\mu }, \end{aligned}$$
  where $D_{SV_{1}V_{2}}$ is a combination of (some of the) parameters entering Lagrangian (16). Then the decay width reads
  43 $$\begin{aligned} \Gamma _{S\rightarrow V_{1}V_{2}}= & {} \mathcal {I}\frac{|{k}|}{4\pi m_{S} ^{2}}D_{SV_{1}V_{2}}^{2}\nonumber \\&\times \,\left[ \frac{(m_{S}^{2}-m_{V_{1}}^{2}-m_{V_{2}} ^{2})^{2}}{8m_{V_{1}}^{2}m_{V_{2}}^{2}}+1\right] . \end{aligned}$$

As is evident from Appendix A, the most general interaction Lagrangian for 3-body decays of the form $S\rightarrow S_{1}S_{2}S_{3}$ is

44 $$\begin{aligned} \mathcal {L}_{SS_{1}S_{2}S_{3}}&=D_{SS_{1}S_{2}S_{3}}\,SS_{1}S_{2} S_{3}+E_{SS_{1}S_{2}S_{3}}\,S(\partial _{\mu }S_{1}\partial ^{\mu }S_{2} )S_{3}\nonumber \\&\quad +(\text {analogous terms with derivative couplings}\nonumber \\&\quad \text {among final states only}). \end{aligned}$$

The ensuing formula for the decay width reads

**Table 3:** Masses of the excited states present in the model. Masses marked with an asterisk are used as input. There is mass degeneracy of $\sigma _N^E$ and $a_0^E$ because we have discarded large-$N_c$ suppressed parameters in our excited-state Lagrangian (16) – see Sect. 2.3.2. The degeneracy of $\eta _N^E$ and $\pi ^E$ is a feature of the model

Model state	$IJ^{P}$	Mass (MeV)	Note
$\sigma _{N}^{E}$	$00^+$	$1790 \pm 35$*	Assigned to $f_0(1790)$
$\eta _{N}^{E}$	$00^-$	$1294 \pm \, \,4$*	Assigned to $\eta (1295)$
$\eta _{S}^{E}$	$00^-$	$1432 \pm 10$*	Assigned to $\eta (1440)$
$\sigma _{S}^{E}$	$00^+$	$1961 \pm 38$	Possible overlap with $f_{0}(2020)$ or $f_0(2100)$
$$ a_{0}^{E}$$	$$10^+$$	$$1790 \pm 35$$	Possible overlap with $$a_{0}(1950)$$
$K_{0}^{\star E}$	$\frac{1}{2} 0^+$	$1877 \pm 36$	Possible overlap with $K_{0}^{\star }(1950)$
$\pi ^{E}$	$10^-$	$1294 \pm \, \, \,4$	Possible overlap with $\pi (1300)$
$K^{E}$	$\frac{1}{2} 0^-$	$1366 \pm \, \, \, 6$	Possible overlap with *K*(1460)

45 $$\begin{aligned} \Gamma _{S\rightarrow S_{1}S_{2}S_{3}}= & {} \mathcal {I}\frac{1}{32(2\pi )^{3} m_{S}^{3}}\int _{(m_{S_{1}}+m_{S_{2}})^{2}}^{(m_{S}-m_{S_{3}})^{2}} \,\nonumber \\&\times \,\text {d}m_{12}^{2}\int _{(m_{23})_{\min .}}^{(m_{23})_{\max .}}\,\text {d} m_{23}^{2}\,\left| \mathcal {M}_{S\rightarrow S_{1}S_{2}S_{3}}\right| ^{2}\; \end{aligned}$$

where $$m_{12}^{2}=(K_{S_{1}}+K_{S_{2}})^{2}$$, $$m_{23}^{2}=(K_{S_{2}}+K_{S_{3} })^{2}$$ and

46 $$\begin{aligned} (m_{23})_{\min .}&=(E_{2}^{*}+E_{3}^{*})^{2}\nonumber \\&\quad -\,\left[ \sqrt{(E_{2}^{*})^{2}-m_{S_{2}}^{2}}+\sqrt{(E_{3}^{*})^{2}-m_{S_{3}}^{2} }\right] ^{2},\end{aligned}$$

47 $$\begin{aligned} (m_{23})_{\max .}&=(E_{2}^{*}+E_{3}^{*})^{2}\nonumber \\&\quad -\,\left[ \sqrt{(E_{2}^{*})^{2}-m_{S_{2}}^{2}}-\sqrt{(E_{3}^{*})^{2}-m_{S_{3}}^{2} }\right] ^{2}, \end{aligned}$$

with

48 $$\begin{aligned} E_{2}^{*}=\frac{m_{12}^{2}-m_{S_{1}}^{2}+m_{S_{2}}^{2}}{2m_{12}} ,\quad E_{3}^{*}=\frac{m_{S}^{2}-m_{12}^{2}-m_{S_{3}}^{2}}{2m_{12}}. \end{aligned}$$

As is evident from Appendix A, our decay widths depend on the following parameters: $g_{1}^{E}$, $\lambda _{2}^{*}$, $\xi _{2}$ and $h_{2,3}^{*}$. The first three appear only in decays with an excited final state; since such decays are experimentally unknown, it is not possible to determine these parameters (and $\xi _{2}$ can be determined from the mass terms in any case; see Sect. 2.3.3). The remaining two, $h_{2,3} ^{*}$, can be calculated from decays with ground states in the outgoing channels – we will discuss this in Sect. 3.3.

## Masses and decays of the excited states: results and consequences

### Parameter determination: general remarks

Combining parameter discussion at the end of Sects. 2.3.3 and 2.3.4, the final conclusion is that the following parameters need to be determined:

49 $$\begin{aligned} C_{1}^{*},C_{2}^{*},\xi _{2},h_{2}^{*}\quad \text { and }\quad h_{3}^{*} \end{aligned}$$

with $C_{1}^{*}$ and $C_{2}^{*}$ parameter combinations defined in Eqs. (28) and (29).

As is evident from mass terms (30)–(36) and Appendix A, $C_{1}^{*}$ and $C_{2}^{*}$ influence only masses; $\xi _{2}$ appears in decays with one excited final state and in mass terms. Since, as indicated at the end of Sect. 2.3.4, decays with excited final states are experimentall unknown, $\xi _{2}$ can only be determined from the masses. Contrarily, $h_{2}^{*}$ and $h_{3}^{*}$ appear only in decay widths (with no excited final states). Hence our parameters are divided in two sets, one determined by masses ($C_{1}^{*}$, $C_{2}^{*}$ and $\xi _{2}$) and another determined by decays (${h_{2}^{\star }}$ and ${h_{3}^{\star }}$).

Parameter determination will ensue by means of a $\chi ^{2}$ fit. Scarcity of experimental data compels us to have an equal number of parameters and experimental data entering the fit; although in that case the equation systems can also be solved exactly, an advantage of the $\chi ^{2}$ fit is that error calculation for parameters and observables is then straightforward.

The general structure of the fit function $\chi ^{2}$ fit is as follows:

50 $$\begin{aligned} \chi ^{2}(p_{1},\ldots ,p_{m})=\sum _{i=1}^{n}\left( \frac{O_{i}^{\text {th.}} (p_{1},\ldots ,p_{m})-O_{i}^{\text {exp.}}}{\Delta O_{i}^{\text {exp.}}}\right) ^{2} \end{aligned}$$

for a set of *n* (theoretical) observables $O_{i}^{\text {th.}}$ determined by $m\le n$ parameters $p_{j}$. In our case, $m=n=3$ for masses and $m=n=2$ for decay widths. Central values and errors on the experimental side are, respectively, denoted $O_{i}^{\text {exp.}}$ and $\Delta O_{i}^{\text {exp.}}$. Parameter errors $\Delta p_{i}$ are calculated as the square roots of the diagonal elements of the inverse Hessian matrix obtained from $\chi ^{2}(p_{j})$. Theoretical errors $\Delta O_{i}$ for each observable $O_{i}$ are calculated by diagonalising the Hesse matrix via a special orthogonal matrix *M*

51 $$\begin{aligned} MHM^{t}\equiv \text {diag}\{\text {eigenvalues of }H\} \end{aligned}$$

and rotating parameters $p_{i}$ such that

52 $$\begin{aligned} {q}=M({p}-{p}_{\min .}) \end{aligned}$$

where *p* contains all parameters and ${p}_{\min .}$ realises the minimum of $\chi ^{2}(p_{1},\ldots ,p_{m})$. Then we can determine $\Delta O_{i}$ via

53 $$\begin{aligned} \Delta O_{i}=\sqrt{\sum _{j=1}^{n}\left( \left. \frac{\partial O_{i} (q_{1},\ldots q_{m})}{\partial q_{j}}\right| _{\text {at fit value of }O_{i} }\Delta q_{j}\right) ^{2}} \end{aligned}$$

(see also Chapter 39 of the Particle Data Book [5]).

### Masses of the excited states

Following the discussion of the experimental data on excited states in Sect. 2.3.1 and particle assignment in Table 2, we use the following masses for the $\chi ^{2}$ fit of Eq. (50): $m_{\sigma _{N}^{E}}\equiv m_{f_{0}(1790)}=(1790\pm 35)$ MeV, $m_{\eta _{N}^{E} }\equiv m_{\eta (1295)}=(1294\pm 4)$ MeV and $m_{\eta _{S}^{E}}\equiv m_{\eta (1440)}=(1432\pm 10)$ MeV. Results for $C_{1}^{*}$, $C_{2}^{*}$ and $\xi _{2}$ are

54 $$\begin{aligned}&C_{1}^{*} =(2.4\pm 0.6)\cdot 10^{6}\text { [MeV}^{2}],\nonumber \\&\quad C_{2}^{*}=(2.5\pm 0.2)\cdot 10^{5}\text { [MeV}^{2}], \quad \xi _{2}=57\pm 5 . \end{aligned}$$

With these parameters, the general discussion from Sect. 3.1 allows us to immediately predict the masses of $\sigma _S^E$, $a_0^E$, $K_0^{\star E}$, $\pi ^E$ and $K^E$. They are presented in Table 3.

**Table 4:** Decays and masses of the excited $\bar{q}q$ states. Widths marked as “suppressed” depend only on large-$N_c$ suppressed parameters that have been set to zero. Widths marked with an asterisk are used as input; the others are predictions

Model state	$IJ^P$	Mass (MeV)	Decay	Width (MeV)	Note
$\sigma _{N}^{E}$	$00^+$	$1790 \pm 35$	$\sigma _N^E \rightarrow \pi \pi$	$270 \pm 45$*	Assigned to $f_0(1790)$; mass, $\pi \pi$ and *KK* decay widths from Ref. [131]. Other decays not (yet) measured
			$\sigma _N^E \rightarrow KK$	$70 \pm 40$*	
			$\sigma _N^E \rightarrow a_1(1260) \pi$	$47 \pm 8$	
			$\sigma _N^E \rightarrow \eta \eta ^{\prime }$	$10 \pm 2$	
			${{\sigma }}_{{N}}^{{E}} {{\rightarrow \eta \eta }}$	${{7 \pm 1}}$	
			$\sigma _N^E \rightarrow f_1(1285) \eta$	$1 \pm 0$	
			$\sigma _N^E \rightarrow K_1 K$	0	
			$\sigma _N^E \rightarrow \sigma _N \pi \pi$	0	
			Total	${405 \pm 96}$	
$\eta _{N}^{E}$	$00^-$	$1294 \pm 4$	$\eta _N^E \rightarrow \eta \pi \pi + \eta ^{\prime } \pi \pi + \pi KK$	$7 \pm 3$	Assigned to $\eta (1295)$; PDG mass [5]
$\eta _{S}^{E}$	$00^-$	$1432 \pm 10$	$\eta _{S}^{E} \rightarrow K^{\star } K$	$128 ^{+204}_{-128}$	Assigned to $\eta (1440)$; mass from Refs. [199, 200]. Full width $\sim$ 100 MeV at this mass [200]. $\Gamma _{\eta (1440) \rightarrow \eta \pi \pi }$ suppressed [200]
			$\eta _{S}^{E} \rightarrow KK \pi$	$28 ^{+41}_{-28}$	
			$\eta _{S}^{E} \rightarrow \eta \pi \pi$ and $\eta ^{\prime } \pi \pi$	Suppressed	
			Total	$$ 156 ^{+245}_{-156}$$	
$$\sigma _{S}^{E}$$	$$00^+$$	$$1961 \pm 38$$	$$\sigma _S^E \rightarrow KK$$	$$21 ^{+39}_{-21} $$	Candidate states: $$f_0(2020)$$; $$m_{f_0(2020)} = (1992 \pm 16)$$ MeV and $$\Gamma _{f_0(2020)} = (442 \pm 60)$$ MeV and $$f_0(2100)$$; $$m_{f_0(2100)} = (2101 \pm 7)$$ MeV and $$\Gamma _{f_0(2101)} = 224^{+23}_{-21} $$ MeV. Both require confirmation [5]
			${\sigma }_{{S}}^{{E}} {{\rightarrow \eta \eta }}^{{\prime }}$	${12} \pm 2$	
			${\sigma }_{{S}}^{{E}} {\rightarrow \eta \eta }$	${ 6 \pm 1 }$	
			$$\sigma _S^E \rightarrow K_1 K$$	$$ 2^{+5}_{-2}$$	
			${\sigma }_{{S}}^{{E}} {{\rightarrow \eta }}^{{\prime }} {\eta }^{{\prime }}$	${1 \pm 0}$	
			$\sigma _S^E \rightarrow \pi \pi$, $\rho \rho$ and $\omega \omega$	Suppressed	
			$\sigma _S^E \rightarrow a_1(1260) \pi$ and $f_1(1285) \eta$	Suppressed	
			$\sigma _S^E \rightarrow \pi ^E \pi$ and $\eta _N^E \eta$	Suppressed	
			$\sigma _S^E \rightarrow \sigma _S \pi \pi$	Suppressed	
			Total	$${42 }^{{+47}}_{{-26}} $$	
$a_{0}^{E}$	$10^+$	$1790 \pm 35$	$a_{0}^{E} \rightarrow \eta \pi$	$72 \pm 12$	Candidate state: $a_0(1950)$; $m_{a_0(1950)} = (1931 \pm 26)$ MeV and $\Gamma _{a_0(1950)} = (271 \pm 40)$ MeV [187]. Requires confirmation [5]
			$a_{0}^{E} \rightarrow KK$	$70 \pm 40$	
			$a_{0}^{E} \rightarrow \eta ^{\prime } \pi$	$32 \pm 5$	
			$a_{0}^{E} \rightarrow f_1(1285)\pi$	$16 \pm 3$	
			$a_{0}^{E} \rightarrow a_1(1260) \eta$	$1 \pm 0$	
			$a_{0}^{E} \rightarrow K_1 K$	0	
			$a_{0}^{E} \rightarrow a_0(1450) \pi \pi$	0	
			Total	$191 \pm 60$	
$K_{0}^{\star E}$	$\frac{1}{2}0^+$	$1877 \pm 36$	$K_{0}^{\star E}\rightarrow K \pi$	$51 \pm 35$	Candidate state: $K_0^{\star }(1950)$; $m_{K_0^{\star }(1950)} = (1945 \pm 22)$ MeV and $\Gamma _{K_0^{\star }(1950)} = (201 \pm 90)$ MeV. Requires confirmation [5]
			${{K}}_{0}^{{\star E}} \rightarrow {{\eta }}^{\prime } {{K}}$	${{24}} {{\pm 4}}$	
			${{K}}_{0}^{{{\star E}}} \rightarrow {{K}}_{{1}} \pi$	${{6 \pm 4}}$	
			$$K_{0}^{\star E} \rightarrow \eta K$$	$$ 4^{+7}_{-4}$$	
			$K_{0}^{\star E} \rightarrow a_1(1260) K$	$3 \pm 2$	
			$K_{0}^{\star E} \rightarrow f_1(1285) K$	$1 \pm 1$	
			$K_{0}^{\star E} \rightarrow K_1 \eta$	0	
			$K_{0}^{\star E} \rightarrow K_0^{\star }(1430) \pi \pi$	0	
			Total	${89} ^{{+53}}_{{-50}}$	
$\pi ^{E}$	$10^-$	$1294 \pm 4$	–	–	Width badly defined due to large errors of the experimental input data
$K^{E}$	$\frac{1}{2}0^-$	$1366 \pm 6$	–	–	Width badly defined due to large errors of the experimental input data

### Decays of the excited states

#### Hypothesis: $f_0(1790)$ is an excited ${\bar{q}}q$ state

We have concluded in Sect. 3.1 that only two parameters are of relevance for all decays predictable in the model: $h_{2}^{*}$ and $h_{3}^{*}$. They can be determined from the data on the $f_0(1790)$ resonance discussed in Sect. 2.3.1: $\Gamma _{f_{0}(1790)\rightarrow \pi \pi }=(270\pm 45)$ MeV and $\Gamma _{f_{0}(1790)\rightarrow KK}=(70\pm 40)$ MeV [131]. Performing the $\chi ^2$ fit described in Sect. 3.1 we obtain the following parameter values:

55 $$\begin{aligned} h_{2}^{*} = 67 \pm 63,\quad h_{3}^{*} = 79 \pm 63. \end{aligned}$$

Large uncertainties for parameters are a consequence of propagation of the large errors for $\Gamma _{f_{0}(1790)\rightarrow \pi \pi }$ and particularly for $\Gamma _{f_{0}(1790)\rightarrow KK}$. As described in Sect. 2.3.1, $\Gamma _{f_{0}(1790)\rightarrow KK}$ was obtained as our estimate relying upon $J/\Psi$ branching ratios reported by BES II [131] that themselves had uncertainties between $\sim$23 and 50%. We emphasise, however, that such uncertainties do not necessarily have to translate into large errors for the observables. The reason is that error calculation involves derivatives at central values of parameters [see Eq. (53)]; small values of derivatives may then compensate the large parameter uncertainties. This is indeed what we observe for most decays.

**Table 5:** Decays and masses for the case where $\eta (1295)$ and $\eta (1440)$ are enforced as excited ${\bar{q}}q$ states. Widths marked with an asterisk were used as input. Pseudoscalar observables compare fine with experiment but the scalars are unobservable due to extremely broad decays into vector mesons

Model state	$IJ^P$	Mass (MeV)	Decay	Width (MeV)	Note
$\eta _{N}^{E}$	$00^-$	$1294 \pm 4$	$\eta _N^E \rightarrow \eta \pi \pi + \eta ^{\prime } \pi \pi + \pi KK$	$55 \pm 5$*	Assigned to $\eta (1295)$; PDG mass [5]
$\eta _{S}^{E}$	$00^-$	$1432 \pm 10$	$\eta _{S}^{E} \rightarrow K^{\star } K$	$26 \pm 3$*	Assigned to $\eta (1440)$; mass and $K^{\star } K$ width from Refs. [199, 200]. Our estimate for $\Delta \Gamma _{\eta (1440) \rightarrow K^{\star } K}$
			$\eta _{S}^{E} \rightarrow KK \pi$	$3 \pm 0$	
			$\eta _{S}^{E} \rightarrow \eta \pi \pi$ and $\eta ^{\prime } \pi \pi$	Suppressed	
			Total	$29 \pm 3$	
$\pi ^{E}$	$10^-$	$1294 \pm 4$	$\pi ^E \rightarrow \rho \pi$	$368 \pm 37$	Assigned to $\pi (1300)$; degenerate in mass with $\eta (1295)$ according to Eq. (32). Compares well with $\Gamma _{\pi (1300)} = (200-600)$ MeV [5]
			$\pi ^E \rightarrow 3\pi$	$204 \pm 15$	
			$\pi ^E \rightarrow K K \pi$	$2 \pm 0$	
			Total	${{574}} \pm 52$	
$K^{E}$	$\frac{1}{2}0^-$	$1366 \pm 6$	${{K}}^{{E}} {{\rightarrow K}}^{{\star }} {{\pi }}$	${{112 \pm 11}}$	Assigned to *K*(1460); $m_{K(1460)} \sim 1460$ MeV; $\Gamma _{K(1460)} \sim 260$ MeV [5]
			$K^{E} \rightarrow K \pi \pi$	$35 \pm 4$	
			$K^{E} \rightarrow \rho K$	$20 \pm 2$	
			$K^E \rightarrow \omega K$	$7 \pm 1$	
			$K^{E} \rightarrow K \pi \eta$	0	
			Total	${{174 \pm 18}}$	
All scalars	–	As in Table 3	See Appendix A	Calculated via Eqs. (39), (41), (43), (45) and Eq. (53)	Unobservable due to extremely large decays into vectors [$\mathcal O$(1 GeV)]

There is a large number of decays that can be calculated using the interaction Lagrangians in Appendix A, parameter values in Eq. (55), formulae for decay widths in Eqs. (39), (41), (43) and (45) as well as Eq. (53) for the errors of observables. All results are presented in Table 4.

The consequences of $f_0(1790)$ input data are then as follows:

- The excited states are generally rather narrow with the exception of $f_0(1790)$ and $\eta (1440)$ whose full decay widths, considering the errors, are, respectively, between $\sim$300 and $\sim$500 MeV and up to $\sim$400 MeV. The result for $f_0(1790)$ is congruent with the data published by LHCb [132]; the large interval for the $\eta (1440)$ width is a consequence of parameter uncertainties, induced by ambiguities in the experimental input data.
- The excited pion and kaon states are also very susceptible to parameter uncertainties that lead to extremely large errors for the $\pi ^E$ and $K^E$ decay widths [$\mathcal O$(1 GeV)]. A definitive statement on these states is therefore not possible. Contrarily, in the case of $\eta (1295)$, the three decay widths accessible to our model (for $\eta _N^E \rightarrow \eta \pi \pi + \eta ^{\prime } \pi \pi + \pi KK$) amount to $(7 \pm 3)$ MeV and hence contribute very little to the overall decay width $\Gamma _{\eta (1295)}^{\text {total}} = (55 \pm 5)$ MeV.
- Analogously to the above point, parameter uncertainties also lead to extremely large width intervals for the decays of scalars into vectors. These decays are therefore omitted from Table 4, except for the large-$N_c$ suppressed decays $\sigma _S^E \rightarrow \rho \rho$ and $\sigma _S^E \rightarrow \omega \omega$.
- Notwithstanding the above two points, we are able to predict more than 35 decay widths for all states in our model except $\pi ^E$ and $K^E$. The overall correspondence of the model states to the experimental (unconfirmed) ones is generally rather good, although we note that our scalar $\bar{s}s$ state appears to be too narrow to fully accommodate either of the $f_0(2020)$ and $f_0(2100)$ states. The mass of our isotriplet state $a_0^E$ is also somewhat smaller than that of $a_0(1950)$ – we will come back to this point in Sect. 3.3.3.

**Fig. 1:**
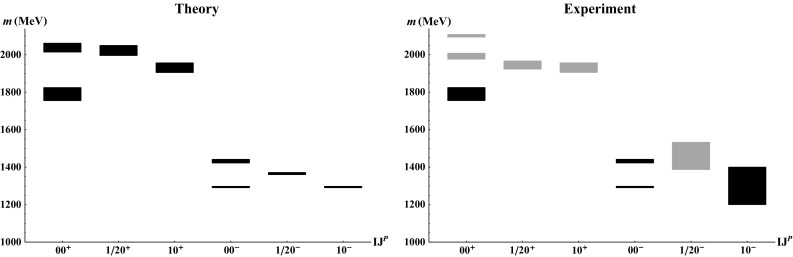
Masses of excited ${\bar{q}}q$ states with isospin *I*, total spin *J* and parity *P* from the Extended Linear Sigma Model (*left*) and masses from the experimental data (*right*). Area *thickness* corresponds to mass uncertainties on both panels. The lower $00^+ (\equiv \sigma _N^E)$, both $00^-(\equiv \eta _N^E$ and $\eta _S^E)$ as well as the $10^+ (\equiv a_0^E)$ states from the *left panel* were used as input. *Lightly shaded areas* correspond to experimentally as yet unestablished states. Table 6 contains the experimental assignment of the states on the left panel and a brief overview of their dynamics

#### Hypothesis: $\eta (1295)$ and $\eta (1440)$ are excited ${\bar{q}}q$ states

As indicated above, results presented in Table 4 do not allow us to make a definitive statement on all excited pseudoscalars. However, the situation changes if the parameters $h_2^{*}$ and $h_3^{*}$ are determined with the help of the $\eta (1295)$ and $\eta (1440)$ decay widths.

Using $\Gamma _{\eta _N^E \rightarrow \eta \pi \pi + \eta ^{\prime } \pi \pi + \pi KK} = (55 \pm 5)$ MeV [5] and $\Gamma _{\eta (1440) \rightarrow K^{\star }K} = 26 \pm 3$ MeV (from Ref. [199]; our estimate for the error) we obtain

56 $$\begin{aligned} h_{2}^{*} = 70 \pm 2,\quad h_{3}^{*} = 35 \pm 3 . \end{aligned}$$

The parameters (56) are strongly constrained and there is a very good correspondence of the pseudoscalar decays to the experimental data in this case (see Table 5). Nonetheless, there is a drawback: all scalar states become unobservable due to very broad decays into vectors. Thus comparison of Tables 4 and 5 suggests that there is tension between the simultaneous interpretation of $\eta (1295)$, $\pi (1300)$, $\eta (1440)$ and *K*(1460) as well as the scalars as excited ${\bar{q}}q$ states. A possible theoretical reason is that pseudoscalars above 1 GeV may have non-${\bar{q}} q$ admixture. Indeed sigma-model studies in Refs. [37, 41, 43, 204–208] have concluded that excited pseudoscalars with masses between 1 GeV and 1.5 GeV represent a mixture of ${\bar{q}}q$ and ${\bar{q}}{\bar{q}}qq$ structures. In addition, the flux-tube model of Ref. [202] and a mixing formalism based on the Ward identity in Ref. [209] lead to the conclusion that the pseudoscalar channel around 1.4 GeV is influenced by a glueball contribution. Hence a more complete description of these states would require implementation of mixing scenarios in this channel.^4^

Note, however, that the results of Table 5 depend on the assumption that the total decay width of $\eta (1295)$ is saturated by the three decay channels accessible to our model ($\eta \pi \pi$, $\eta ^{\prime } \pi \pi$ and $K \pi \pi$). The level of justification for this assumption is currently uncertain [5]. Consequently we will not explore this scenario further.

#### Is $a_0(1950)$ of the BABAR Collaboration an excited ${\bar{q}}q$ state?

Encouraging results obtained in Sect. 3.3.1, where $f_0(1790)$ was assumed to be an excited ${\bar{q}}q$ state, can be used as a motivation to explore them further. As discussed in Sect. 2.3.1, data analysis published recently by the BABAR Collaboration has found evidence of an isotriplet state $a_0(1950)$ with mass $m_{a_0(1950)} = (1931 \pm 26)$ MeV and decay width $\Gamma _{a_0(1950)} = (271 \pm 40)$ MeV [187].

Assuming that $f_0(1790)$ is an excited ${\bar{q}}q$ state (as already done in Sect. 3.3.1), we can implement $m_{a_0(1950)}$ obtained by BABAR as a large-$N_c$ suppressed effect in our model as follows. Mass terms for excited states $\sigma _N^E$ and $\sigma _S^E$, Eqs. (30) and (34), can be modified by reintroduction of the large-$N_c$ suppressed parameter $\kappa _2$ and now read

57 $$\begin{aligned}&m_{\sigma _{N}^{E}}^{2} =C_{1}^{*}+\left( \frac{\xi _{2}}{2}+ 2 \kappa _2 \right) \phi _{N} ^{2},\end{aligned}$$

58 $$\begin{aligned}&m_{\sigma _{S}^{E}}^{2} =C_{1}^{*}+2C_{2}^{*}+\left( \frac{\xi _{2}}{2}+ 2 \kappa _2 \right) (\phi _{N}-2Z_{K}f_{K})^{2}. \end{aligned}$$

**Table 6:** Final results: decays and masses of the excited $\bar{q}q$ states. Widths marked as “suppressed” depend only on large-$N_c$ suppressed parameters that have been set to zero. Masses/widths marked with (*) are used as input; others are predictions

Model state	$IJ^P$	Mass (MeV)	Decay	Width (MeV)	Note
$\sigma _{N}^{E}$	$00^+$	$1790 \pm 35$*	$\sigma _N^E \rightarrow \pi \pi$	$270 \pm 45$*	Assigned to $f_0(1790)$; mass, $\pi \pi$ and *KK* decay widths fixed to BES II data [131]. Other decays not (yet) measured
			$\sigma _N^E \rightarrow KK$	$70 \pm 40$*	
			$\sigma _N^E \rightarrow a_1(1260) \pi$	$47 \pm 8$	
			$\sigma _N^E \rightarrow \eta \eta ^{\prime }$	$10 \pm 2$	
			${\sigma }_{{N}}^{{E}} {{\rightarrow \eta \eta }}$	${7 \pm 1}$	
			$\sigma _N^E \rightarrow f_1(1285) \eta$	$1 \pm 0$	
			$\sigma _N^E \rightarrow K_1 K$	0	
			$\sigma _N^E \rightarrow \sigma _N \pi \pi$	0	
			Total	${405 \pm 96}$	
$a_{0}^{E}$	$10^+$	$1931 \pm 26$*	$a_{0}^{E} \rightarrow \eta \pi$	$94 \pm 16$	Candidate state: $a_0(1950)$ recently measured by BABAR; $m_{a_0(1950)} = (1931 \pm 26)$ MeV and $\Gamma _{a_0(1950)} = (271 \pm 40)$ MeV [187] Requires confirmation [5]
			$a_{0}^{E} \rightarrow KK$	$94 \pm 54$	
			$a_{0}^{E} \rightarrow \eta ^{\prime } \pi$	$48 \pm 8$	
			$a_{0}^{E} \rightarrow f_1(1285) \pi$	$28 \pm 5$	
			$a_{0}^{E} \rightarrow K_1 K$	$9 \pm 5$	
			$a_{0}^{E} \rightarrow a_1(1260) \eta$	$6 \pm 1$	
			$a_{0}^{E} \rightarrow a_0(1450) \pi \pi$	$1 \pm 1$	
			Total	$280 \pm 90$	
$\eta _{N}^{E}$	$00^-$	$1294 \pm 4$*	$\eta _N^E \rightarrow \eta \pi \pi + \eta ^{\prime } \pi \pi + \pi KK$	$7 \pm 3$	Assigned to $\eta (1295)$; PDG mass [5]
$$\eta _{S}^{E}$$	$$00^-$$	$$1432 \pm 10$$*	$$\eta _{S}^{E} \rightarrow K^{\star } K$$	$$ 128 ^{+204}_{-128}$$	Assigned to $$\eta (1440)$$; mass from BES data [199, 200]. Full width $$\sim $$ 100 MeV at this mass [200]. $$\Gamma _{\eta (1440) \rightarrow \eta \pi \pi }$$ suppressed [200]
			$\eta _{S}^{E} \rightarrow KK \pi$	$28 ^{+41}_{-28}$	
			$\eta _{S}^{E} \rightarrow \eta \pi \pi$ and $\eta ^{\prime } \pi \pi$	Suppressed	
			Total	$$ 156 ^{+245}_{-156}$$	
$\sigma _{S}^{E}$	$00^+$	$2038 \pm 24$	$\sigma _S^E \rightarrow KK$	$24 ^{+46}_{-24}$	Candidate states: $f_0(2020)$; $m_{f_0(2020)} = (1992 \pm 16)$ MeV and $\Gamma _{f_0(2020)} = (442 \pm 60)$ MeV and $f_0(2100)$; $m_{f_0(2100)} = (2101 \pm 7)$ MeV and $\Gamma _{f_0(2101)} = 224^{+23}_{-21}$ MeV. Both require confirmation [5]
			$\sigma _S^E \rightarrow \eta \eta ^{\prime }$	$16 \pm 3$	
			${\sigma }_{{S}}^{{E}} {{\rightarrow \eta \eta }}$	${7 \pm 1}$	
			$$\sigma _S^E \rightarrow K_1 K$$	$$ 4^{+8}_{-4}$$	
			${\sigma }_{{S}}^{{E}} {{\rightarrow \eta }}^{{\prime }} {{\eta }}^{{\prime }}$	${{ 1 \pm 0}}$	
			$\sigma _S^E \rightarrow \pi \pi$, $\rho \rho$ and $\omega \omega$	Suppressed	
			$\sigma _S^E \rightarrow a_1(1260) \pi$ and $f_1(1285) \eta$	Suppressed	
			$\sigma _S^E \rightarrow \pi ^E \pi$ and $\eta _N^E \eta$	Suppressed	
			$\sigma _S^E \rightarrow \sigma _S \pi \pi$	Suppressed	
			Total	$${52} ^{{+58}}_{{-32}} $$	
$K_{0}^{\star E}$	$\frac{1}{2}0^+$	$2023 \pm 27\;$	$K_{0}^{\star E} \rightarrow \eta ^{\prime } K$	$72 \pm 12$	Candidate state: $K_0^{\star }(1950)$; $m_{K_0^{\star }(1950)} = (1945 \pm 22)$ MeV and $\Gamma _{K_0^{\star }(1950)} = (201 \pm 90)$ MeV. Requires confirmation [5]
			$K_{0}^{\star E}\rightarrow K \pi$	$66 \pm 46$	
			$K_{0}^{\star E} \rightarrow K_1 \pi$	$10 \pm 7$	
			${K}_{{0}}^{{\star E}} {\rightarrow a}_{{1}}{{(1260)}} {{K}}$	${{6 \pm 4}}$	
			$$K_{0}^{\star E} \rightarrow \eta K$$	$$ 6^{+9}_{-6}$$	
			${K}_{{0}}^{{\star E}} {\rightarrow f}_{{1}}{{(1285)}} {{K}}$	${{2}} \pm 1$	
			$K_{0}^{\star E} \rightarrow K_1 \eta$	0	
			$K_{0}^{\star E} \rightarrow K_0^{\star }(1430) \pi \pi$	0	
			Total	${162} ^{{+79}}_{{-76}}$	
$\pi ^{E}$	$10^-$	$1294 \pm 4$	–	–	Width badly defined due to large errors of the experimental input data
$K^{E}$	$\frac{1}{2}0^-$	$1366 \pm 6$	–	–	Width badly defined due to large errors of the experimental input data

The other mass terms [Eqs. (31)–(33), (35) and (36)] remain exactly the same; $\kappa _2$ does not influence any decay widths. We can now repeat the calculations described in Sect. 3.2 with the addition that the mass of our state $a_0^E$ corresponds exactly to that of $a_0(1950)$. We obtain

59 $$\begin{aligned}&C_{1}^{*} =(2.4\pm 0.6)\cdot 10^{6}\text { [MeV}^{2}],\quad C_{2}^{*}\nonumber \\&\quad =(2.5\pm 0.2)\cdot 10^{5}\text { [MeV}^{2}],\nonumber \\&\qquad \xi _{2}=57\pm 5, \kappa _2 = -10 \pm 3. \end{aligned}$$

Note that a non-vanishing value of $\kappa _2$ introduces mixing of $\sigma _N^E$ and $\sigma _S^E$ in our Lagrangian (16). Its effect is, however, vanishingly small since the mixing angle is $\sim 11^{\circ }$.

Using the mass parameters (59) and the decay parameters (55) we can repeat the calculations of Sect. 3.3.1. Then our final results for the mass spectrum are presented in Fig. 1 and for the decays in Table 6. The values of $m_{a_0^E}$, $m_{\sigma _S^E}$ and $m_{K_0^{\star E}}$ have changed in comparison to Table 4 inducing an increased phase space. For this reason, the decay widths of the corresponding resonances have changed as well. All other results from Table 4 have remained the same and are again included for clarity and convenience of the reader.

The consequences are as follows:

- The decay width of $a_0^E$ is now $\Gamma _{a_0^E} = (280 \pm 90)$ MeV; it overlaps fully with $\Gamma _{a_0(1950)} = (271 \pm 40)$ MeV measured by BABAR. Hence, if $a_0(1950)$ is confirmed in future measurements, it will represent a very good candidate for the excited isotriplet $\bar{n}n$ state.
- The mass of $\sigma _S^E$ is between those of $f_0(2020)$ and $f_0(2100)$. Judging by the quantum numbers, either of these resonances could represent a (predominant) $\bar{s}s$ state; an option is also that the excited $\bar{s}s$ state with $IJ^{PC} = 00^{++}$ has not yet been observed in this energy region. However, one must also remember the possibility that ${\bar{q}}q$–glueball mixing (neglected here) may change masses as well as decay patterns. The decay width of $\sigma _S^E$ is rather narrow (up to 110 MeV) but this may change if mixing effects happen to be large.
- The mass of $K_{0}^{\star E}$ is qualitatively (within $\sim$ 100 MeV) congruent with that of $K_{0}^{\star } (1950)$; the widths overlap within 1 $\sigma$. Hence, if $K_{0}^{\star } (1950)$ is confirmed in future measurements, it will represent a very good candidate for the excited scalar kaon.
- Conclusions for all other states remain as in Sect. 3.3.1.

## Conclusion

We have studied masses and decays of excited scalar and pseudoscalar ${\bar{q}}q$ states ($q = u,d,s$ quarks) in the Extended Linear Sigma Model (eLSM) that, in addition, contains ground-state scalar, pseudoscalar, vector and axial-vector mesons.

Our main objective was to study the assumption that the $f_0(1790)$ resonance is an excited $\bar{n}n$ state. This assignment was motivated by the observation in BES [131] and LHCb [132] data that the resonance couples mostly to pions and by the theoretical statement that the $\bar{n}n$ ground state is contained in the physical spectrum below $f_0(1790)$. Furthermore, the assumption was also tested that the $a_0(1950)$ resonance, whose discovery was recently claimed by the BABAR Collaboration [187], represents the isotriplet partner of $f_0(1790)$.

Using the mass, $2\pi$ and 2*K* decay widths of $f_0(1790)$, the mass of $a_0(1950)$ and the masses of the pseudoscalar isosinglets $\eta (1295)$ and $\eta (1440)$ our model predicts more than 35 decays for all excited states except for the excited pion and kaon (where extremely large uncertainties are present due to experimental ambiguities). All numbers are collected in Table 6.

In essence: the $f_0(1790)$ resonance emerges as the broadest excited ${\bar{q}}q$ state in the scalar channel with ${\Gamma _{f_0(1790)}} = (405 \pm 96)$ MeV; $a_0(1950)$, if confirmed, represents a very good candidate for the excited ${\bar{q}}q$ state; $K_0^{\star }(1950)$, if confirmed, represents a very good candidate for the excited scalar kaon.

Our excited isoscalar $\bar{s}s$ state has a mass of $(2038 \pm 24)$ MeV, placed between the masses of the nearby $f_0(2020)$ and $f_0(2100)$ resonances; also, its width is relatively small (${\le 110}$ MeV). We conclude that, although any of these resonances may in principle represent a ${\bar{q}}q$ state, the introduction of mixing effects (particularly with a glueball state) may be necessary to further elucidate their structure.

Our results also imply a quite small contribution of the $\eta \pi \pi$, $\eta ^{\prime } \pi \pi$ and $\pi KK$ decays to the overall width of $\eta (1295)$. For $\eta (1440)$, the decay width is compatible with any value up to $\sim 400$ MeV (ambiguities due to uncertainty in experimental input data).

It is also possible to implement $\Gamma _{\eta (1295)}^{\text {total}} \equiv \Gamma _{\eta (1295) \rightarrow \eta \pi \pi } {}_{+ \eta ^{\prime } \pi \pi + \pi KK}$ and $\Gamma _{\eta (1440) \rightarrow K^{\star }K}$ exactly as in the data of PDG [5] and BES [199]. Then $\pi (1300)$ and *K*(1460) are quite well described as excited ${\bar{q}}q$ states – but the scalars are unobservably broad (see Table 5). Hence, in this case, there appears to be tension between the simultaneous description of $\eta (1295)$, $\pi (1300)$, $\eta (1440)$ and *K*(1460) and their scalar counterparts as excited ${\bar{q}}q$ states. This scenario is, however, marred by experimental uncertainties: for example, it is not at all clear if the width of $\eta (1295)$ is indeed saturated by the $\eta \pi \pi$, $\eta ^{\prime } \pi \pi$ and $\pi KK$ decays. It could therefore only be explored further when (very much needed) new experimental data arrives – from BABAR, BES, LHCb or PANDA [81] and NICA [212].

## Appendix A: Interaction Lagrangians

Here we collect all interaction Lagrangians that are used for calculations of decay widths throughout this article. Vertices for large-$N_{c}$ suppressed decays are not included but briefly discussed after each Lagrangian in which they appear.

Appendix A.1: Lagrangian for $\sigma _N^E$

The Lagrangian reads

A.1 $$\begin{aligned}&\mathcal {L}_{\sigma _{N}^{E}} =\,\frac{1}{2}(h_{2}^{\star }-h_{3}^{\star })w_{a_{1}}^{2}Z_{\pi }^{2}\phi _{N}\,\sigma _{N}^{E}\left[ (\partial _{\mu } \eta _{N})^{2}+(\partial _{\mu }\mathbf {\pi })^{2}\right] \nonumber \\&\qquad \qquad +\,\frac{1}{2}\left( h_{2}^{\star }\phi _{N}-\sqrt{2}h_{3}^{\star }\phi _{S}\right) w_{K_{1}}^{2} Z_{K}^{2}\,\sigma _{N}^{E}\nonumber \\&\qquad \qquad \times \left( \partial _{\mu }{\bar{K}}^{0}\partial ^{\mu } K^{0}+\partial _{\mu }K^{-}\partial ^{\mu }K^{+}\right) \nonumber \\&\qquad \qquad +\,(h_{2}^{\star }-h_{3}^{\star })w_{a_{1}}Z_{\pi }\phi _{N}\,\sigma _{N} ^{E}\left( f_{1N}^{\mu }\partial _{\mu }\eta _{N}+\mathbf {a_{1}}^{\mu }\cdot \partial _{\mu }\mathbf {\pi }\right) \nonumber \\&\qquad \qquad +\,\frac{1}{2}\left( h_{2}^{\star }\phi _{N}-\sqrt{2}h_{3}^{\star }\phi _{S}\right) w_{K_{1}}Z_{K}\,\sigma _{N}^{E}\nonumber \\&\qquad \qquad \times \, \left( {\bar{K}}_{1\mu }^{0} \partial ^{\mu }K^{0}+K_{1\mu }^{-}\partial ^{\mu }K^{+}\text { + h.c.}\right) \nonumber \\&\qquad \qquad +\,\frac{1}{2}(h_{2}^{\star }+h_{3}^{\star })\phi _{N}\,\sigma _{N}^{E}\left[ (\omega _{N}^{\mu })^{2}+(\mathbf {\rho }^{\mu })^{2}\right] \nonumber \\&\qquad \qquad +\,\frac{1}{2}\left( h_{2}^{\star }\phi _{N}+\sqrt{2}h_{3}^{\star }\phi _{S}\right) \,\sigma _{N} ^{E}\left( {\bar{K}}_{\mu }^{\star 0}K^{\star \mu 0}+K_{\mu }^{\star -}K^{\star \mu +}\right) \nonumber \\&\qquad \qquad -\,\xi _{2}Z_{\pi }\phi _{N}\,\sigma _{N}^{E}\mathbf {\pi }^{E}\cdot \mathbf {\pi } -g_{1}^{E}w_{a_{1}}Z_{\pi }\,\sigma _{N}^{E}\partial _{\mu }\mathbf {\pi }^{E} \cdot \partial ^{\mu }\mathbf {\pi }\nonumber \\&\qquad \qquad +\,\frac{1}{2}(h_{2}^{\star }-h_{3}^{\star } )w_{a_{1}}^{2}Z_{\pi }^{2}\,\sigma _{N}^{E}\sigma _{N}(\partial _{\mu }\mathbf {\pi })^{2}. \end{aligned}$$

Note: the decay $\sigma _{N}^{E}\rightarrow \eta _{S}\eta _{S}$ ($\sim \kappa _{1}$, $h_{1}^{\star }$) is large-$N_{c}$ suppressed.

Appendix A.2: Lagrangian for $\sigma _{\textit{S}}^{\textit{E}}$

The Lagrangian reads

A.2 $$\begin{aligned}&\mathcal {L}_{\sigma _{S}^{E}} =\,(h_{2}^{\star }-h_{3}^{\star })w_{f_{1S} }^{2}Z_{\eta _{S}}^{2}\phi _{S}\,\sigma _{S}^{E}(\partial _{\mu }\eta _{S})^{2} \nonumber \\&\qquad \qquad +\left( h_{2}^{\star }\phi _{S}-\frac{h_{3}^{\star }}{\sqrt{2}}\phi _{N}\right) \nonumber \\&\qquad \qquad \times \,w_{K_{1}}^{2}Z_{K}^{2}\,\sigma _{S}^{E}\left( \partial _{\mu }{\bar{K}} ^{0}\partial ^{\mu }K^{0}+\partial _{\mu }K^{-}\partial ^{\mu }K^{+}\right) \nonumber \\&\qquad \qquad +\,\left( h_{2}^{\star }\phi _{S}-\frac{h_{3}^{\star }}{\sqrt{2}}\phi _{N}\right) w_{K_{1}}Z_{K}\,\sigma _{S}^{E}\nonumber \\&\qquad \qquad \times \,\left( {\bar{K}}_{1\mu }^{0} \partial ^{\mu }K^{0}+K_{1\mu }^{-}\partial ^{\mu }K^{+}\text { + h.c.}\right) \nonumber \\&\qquad \qquad +\,\left( h_{2}^{\star }\phi _{S}+\frac{h_{3}^{\star }}{\sqrt{2}}\phi _{N}\right) \,\sigma _{S}^{E}\left( {\bar{K}}_{\mu }^{\star 0}K^{\star \mu 0}+K_{\mu }^{\star -}K^{\star \mu +}\right) . \end{aligned}$$

Note: the decays $\sigma _{S}^{E}\rightarrow \pi \pi$ ($\sim \kappa _{1}$, $h_{1}^{\star }$), $\sigma _{S}^{E}\rightarrow \eta _{N}\eta _{N}$ ($\sim \kappa _{1}$, $h_{1}^{\star }$), $\sigma _{S}^{E}\rightarrow \rho \rho$ ($\sim h_{1}^{\star }$), $\sigma _{S}^{E}\rightarrow \omega _{N}\omega _{N}$ ($\sim h_{1}^{\star }$), $\sigma _{S}^{E}\rightarrow a_{1}\pi$ ($\sim h_{1}^{\star }$), $\sigma _{S}^{E}\rightarrow f_{1N}\eta _{N}$ ($\sim h_{1}^{\star }$), $\sigma _{S}^{E}\rightarrow \pi ^{E}\pi$ ($\sim \kappa _{2}$), $\sigma _{S}^{E} \rightarrow \eta _{N}^{E}\eta _{N}$ ($\sim \kappa _{2}$) and $\sigma _{S} ^{E}\rightarrow \sigma _{S}\pi \pi$ ($\sim \kappa _{1}$, $h_{1}^{\star }$) are large-$N_{c}$ suppressed.

Appendix A.3: Lagrangian for $\textit{a}_{0}^{\textit{E}}$

The Lagrangian reads (only $a_{0}^{0E}$ included; decays of $a_{0}^{\pm E}$ follow from isospin symmetry):

A.3 $$\begin{aligned}&\mathcal {L}_{a_{0}^{E}} =\,(h_{2}^{\star }-h_{3}^{\star })w_{a_{1}} ^{2}Z_{\pi }^{2}\phi _{N}\,a_{0}^{0E}\partial _{\mu }\pi ^{0}\partial ^{\mu }\eta _{N}\nonumber \\&\qquad \qquad -\,\frac{1}{2}\left( h_{2}^{\star }\phi _{N}-\sqrt{2}h_{3}^{\star }\phi _{S}\right) w_{K_{1}}^{2}Z_{K}^{2}\,a_{0}^{0E}\nonumber \\&\qquad \qquad \times \,\left( \partial _{\mu }\bar{K}^{0}\partial ^{\mu }K^{0}-\partial _{\mu }K^{-}\partial ^{\mu }K^{+}\right) \nonumber \\&\qquad \qquad +\,(h_{2}^{\star }-h_{3}^{\star })w_{a_{1}}Z_{\pi }\phi _{N}\,a_{0}^{0E}\left( f_{1N}^{\mu }\partial _{\mu }\pi ^{0}+a_{1}^{\mu 0}\partial _{\mu }\eta _{N}\right) \nonumber \\&\qquad \qquad -\,\frac{1}{2}\left( h_{2}^{\star }\phi _{N}-\sqrt{2}h_{3}^{\star }\phi _{S}\right) w_{K_{1}}Z_{K}\,a_{0}^{0E}\nonumber \\&\qquad \qquad \times \,\left( {\bar{K}}_{1\mu }^{0}\partial ^{\mu }K^{0}-K_{1\mu }^{-}\partial ^{\mu }K^{+}\text { + h.c.}\right) \nonumber \\&\qquad \qquad +\,(h_{2}^{\star }+h_{3}^{\star })\phi _{N}\,a_{0}^{0E}\rho _{\mu }^{0}\omega _{N}^{\mu }-\frac{1}{2}\left( h_{2}^{\star }\phi _{N}+\sqrt{2}h_{3}^{\star } \phi _{S}\right) \nonumber \\&\qquad \qquad \times \,a_{0}^{0E}\left( {\bar{K}}_{\mu }^{\star 0}K^{\star \mu 0}-K_{\mu }^{\star -}K^{\star \mu +}\right) \nonumber \\&\qquad \qquad -\,\xi _{2}Z_{\pi }\phi _{N}\,a_{0}^{0E}\eta _{N}^{E}\pi ^{0}-g_{1}^{E}w_{a_{1} }Z_{\pi }\,a_{0}^{0E}\partial _{\mu }\eta _{N}^{E}\partial ^{\mu }\pi ^{0}\nonumber \\&\qquad \qquad +\,\frac{1}{2}(h_{2}^{\star }+h_{3}^{\star })w_{a_{1}}^{2}Z_{\pi }^{2} \,a_{0}^{0E}a_{0}^{0}(\partial _{\mu }\mathbf {\pi })^{2}\nonumber \\&\qquad \qquad -\,h_{3}^{\star }w_{a_{1}} ^{2}Z_{\pi }^{2}\,a_{0}^{0E}\partial _{\mu }\pi ^{0}\left( \mathbf {a}_{0} \cdot \partial ^{\mu }\mathbf {\pi }\right) . \end{aligned}$$

Appendix A.4: Lagrangian for $K_{0}^{\star E}$

The Lagrangian reads (only $K_{0}^{\star 0E}$ included; decays of other $K_{0}^{\star E}$ components follow from isospin symmetry):

A.4 $$\begin{aligned}&\mathcal {L}_{K_{0}^{\star E}} =\frac{1}{4}\left[ h_{2}^{\star }\left( \phi _{N}+\sqrt{2}\phi _{S}\right) -2h_{3}^{\star }\phi _{N}\right] w_{a_{1} }w_{K_{1}}Z_{\pi }Z_{K}\,K_{0}^{\star 0E}\nonumber \\&\qquad \qquad \times \,\left( \partial _{\mu }{\bar{K}} ^{0}\partial ^{\mu }\eta _{N}-\partial _{\mu }{\bar{K}}^{0}\partial ^{\mu }\pi ^{0}+\sqrt{2}\partial _{\mu }K^{-}\partial ^{\mu }\pi ^{+}\right) \nonumber \\&\qquad \qquad +\,\frac{1}{2\sqrt{2}}\left[ h_{2}^{\star }\left( \phi _{N}+\sqrt{2}\phi _{S}\right) -2\sqrt{2}h_{3}^{\star }\phi _{S}\right] \nonumber \\&\qquad \qquad \times w_{f_{1S}}w_{K_{1} }Z_{\eta _{S}}\,Z_{K}\,K_{0}^{\star 0E}\partial _{\mu }{\bar{K}}^{0}\partial ^{\mu } \eta _{S}\nonumber \\&\qquad \qquad +\,\frac{1}{4}\left[ h_{2}^{\star }\left( \phi _{N}+\sqrt{2}\phi _{S}\right) -2h_{3}^{\star }\phi _{N}\right] w_{K_{1}}Z_{K}\,K_{0}^{\star 0E}\nonumber \\&\qquad \qquad \times \,\left( f_{1N}^{\mu }\partial _{\mu }{\bar{K}}^{0}-a_{1}^{\mu 0}\partial _{\mu }{\bar{K}} ^{0}+\sqrt{2}a_{1}^{\mu +}\partial _{\mu }K^{-}\right) \nonumber \\&\qquad \qquad +\,\frac{1}{4}\left[ h_{2}^{\star }\left( \phi _{N}+\sqrt{2}\phi _{S}\right) -2h_{3}^{\star }\phi _{N}\right] w_{a_{1}}Z_{\pi }\,K_{0}^{\star 0E}\nonumber \\&\qquad \qquad \times \,\left( {{\bar{K}}}_{1\mu }^{0}\partial ^{\mu }\eta _{N}-{\bar{K}}_{1\mu }^{0}\partial ^{\mu } \pi ^{0}+\sqrt{2}K_{1\mu }^{-}\partial ^{\mu }\pi ^{+}\right) \nonumber \\&\qquad \qquad +\,\frac{1}{2\sqrt{2}}\left[ h_{2}^{\star }\left( \phi _{N}+\sqrt{2}\phi _{S}\right) -2\sqrt{2}h_{3}^{\star }\phi _{S}\right] \nonumber \\&\qquad \qquad w_{f_{1S}}Z_{\eta _{S} }\,K_{0}^{\star 0E}{\bar{K}}_{1\mu }^{0}\partial ^{\mu }\eta _{S}\nonumber \\&\qquad \qquad +\,\frac{1}{4}\left[ h_{2}^{\star }\left( \phi _{N}+\sqrt{2}\phi _{S}\right) +2h_{3}^{\star }\phi _{N}\right] \,K_{0}^{\star 0E}\nonumber \\&\qquad \qquad \times \, \left( {\bar{K}}_{\mu } ^{\star 0}\omega _{N}^{\mu }-{\bar{K}}_{\mu }^{\star 0}\rho ^{\mu 0}+\sqrt{2}K_{\mu }^{\star -}\rho ^{\mu +}\right) \nonumber \\&\qquad \qquad -\,\frac{1}{4}\left[ 2\xi _{2}\phi _{N}-\lambda _{2}^{\star }\left( \phi _{N}-\sqrt{2}\phi _{S}\right) \right] Z_{K}\,K_{0}^{\star 0E}\nonumber \\&\qquad \qquad \times \, \left( \bar{K}^{0}\eta _{N}^{E}-{\bar{K}}^{0}\pi ^{0E}+\sqrt{2}K^{-}\pi ^{+E}\right) \nonumber \\&\qquad \qquad -\,\frac{1}{2}g_{1}^{E}w_{K_{1}}Z_{K}\,K_{0}^{\star 0E}\nonumber \\&\qquad \qquad \times \,\left( \partial _{\mu }{\bar{K}}^{0}\partial ^{\mu }\eta _{N}^{E}-\partial _{\mu }{\bar{K}}^{0}\partial ^{\mu }\pi ^{0E}+\sqrt{2}\partial _{\mu }K^{-}\partial ^{\mu }\pi ^{+E}\right) \nonumber \\&\qquad \qquad +\,\frac{1}{2}g_{1}^{E}w_{K_{1}}Z_{K}\,\partial _{\mu }K_{0}^{\star 0E}\nonumber \\&\qquad \qquad \times \,\left( \partial ^{\mu }{\bar{K}}^{0}\eta _{N}^{E}-\partial ^{\mu }{\bar{K}}^{0}\pi ^{0E} +\sqrt{2}\partial ^{\mu }K^{-}\pi ^{+E}\right) \nonumber \\&\qquad \qquad +\,\frac{1}{\sqrt{2}}\xi _{2}Z_{\pi }\phi _{S}\,K_{0}^{\star 0E}\left( \bar{K}^{0E}\pi ^{0}-\sqrt{2}K^{-E}\pi ^{+}\right) \nonumber \\&\qquad \qquad +\, \frac{1}{2}g_{1}^{E}w_{a_{1} }Z_{\pi }\,K_{0}^{\star 0E}\nonumber \\&\qquad \qquad \times \,\left( \partial _{\mu }{\bar{K}}^{0E}\partial ^{\mu } \pi ^{0}-\sqrt{2}\partial _{\mu }K^{-E}\partial ^{\mu }\pi ^{+}\right) \nonumber \\&\qquad \qquad -\,\frac{1}{2}g_{1}^{E}w_{a_{1}}Z_{\pi }\,\partial _{\mu }K_{0}^{\star 0E}\nonumber \\&\qquad \qquad \times \,\left( {\bar{K}}^{0E}\partial ^{\mu }\pi ^{0}-\sqrt{2}K^{-E}\partial ^{\mu }\pi ^{+}\right) \nonumber \\&\qquad \qquad +\,\frac{1}{4}h_{2}^{\star }w_{a_{1}}^{2}Z_{\pi }^{2}Z_{K_{S}}\,K_{0}^{\star 0E}{\bar{K}}_{0}^{\star 0}(\partial _{\mu }\mathbf {\pi })^{2}\nonumber \\&\qquad \qquad +\,\frac{i}{4}(h_{2}^{\star }-2h_{3}^{\star })w_{a_{1}}w_{K^{\star }}^{*}Z_{\pi }^{2}Z_{K_{S}} \,K_{0}^{\star 0E}\pi ^{0}\partial _{\mu }{\bar{K}}_{0}^{\star 0}\partial ^{\mu } \pi ^{0}\nonumber \\&\qquad \qquad -\,ih_{3}^{\star }w_{a_{1}}w_{K^{\star }}^{*}Z_{\pi }^{2}Z_{K_{S}} \,K_{0}^{\star 0E}\pi ^{-}\partial _{\mu }{\bar{K}}_{0}^{\star 0}\partial ^{\mu } \pi ^{+}\nonumber \\&\qquad \qquad +\,\frac{i}{2}h_{2}^{\star }w_{a_{1}}w_{K^{\star }}^{*}Z_{\pi } ^{2}Z_{K_{S}}\,K_{0}^{\star 0E}\pi ^{+}\partial _{\mu }{\bar{K}}_{0}^{\star 0}\partial ^{\mu }\pi ^{-}\nonumber \\&\qquad \qquad +\,\frac{i}{2\sqrt{2}}(h_{2}^{\star }+2h_{3}^{\star })w_{a_{1}}w_{K^{\star } }^{*}Z_{\pi }^{2}Z_{K_{S}}\,K_{0}^{\star 0E}\nonumber \\&\qquad \qquad \times \,\left( \pi ^{+}\partial _{\mu }K_{0}^{\star -}\partial ^{\mu }\pi ^{0}-\pi ^{0}\partial _{\mu }K_{0}^{\star -}\partial ^{\mu }\pi ^{+}\right) . \end{aligned}$$

Appendix A.5: Lagrangian for $\eta _{N}^{E}$

Only three-body decays into pseudoscalars are kinematically allowed for this particle:

A.5 $$\begin{aligned}&\mathcal {L}_{\eta _{N}^{E}} =\,\frac{1}{2}(h_{2}^{\star }-h_{3}^{\star })w_{a_{1}}^{2}Z_{\pi }^{3}\,\eta _{N}^{E}\eta _{N}(\partial _{\mu }\mathbf {\pi } )^{2}\nonumber \\&\qquad \qquad +\,(h_{2}^{\star }-h_{3}^{\star })w_{a_{1}}^{2}Z_{\pi }^{3}\,\eta _{N} ^{E}\left( \partial _{\mu }\eta _{N}\partial ^{\mu }\mathbf {\pi }\right) \cdot \mathbf {\pi }\nonumber \\&\qquad \qquad -\,\frac{1}{4}(h_{2}^{\star }-2h_{3}^{\star })w_{a_{1}}w_{K_{1}}Z_{\pi } Z_{K}^{2}\,\eta _{N}^{E}\nonumber \\&\qquad \qquad \times \left( {\bar{K}}^{0}\partial _{\mu }K^{0}\partial ^{\mu } \pi ^{0}-\sqrt{2}{\bar{K}}^{0}\partial _{\mu }K^{+}\partial ^{\mu }\pi ^{-} \right. \nonumber \\&\qquad \qquad \left. -\, K^{-}\partial _{\mu }K^{+}\partial ^{\mu }\pi ^{0}-\sqrt{2} K^{-}\partial _{\mu }K^{0}\partial ^{\mu }\pi ^{+}\text { + h.c.}\right) \nonumber \\&\qquad \qquad -\,\frac{1}{2}h_{2}^{\star }w_{K_{1}}^{2}Z_{\pi }Z_{K}^{2}\,\eta _{N}^{E}\nonumber \\&\qquad \qquad \times \left( \pi ^{0}\partial _{\mu }{\bar{K}}^{0}\partial ^{\mu }K^{0}-\pi ^{0}\partial _{\mu } K^{-}\partial ^{\mu }K^{+}\right. \nonumber \\&\qquad \qquad \left. -\sqrt{2}\pi ^{-}\partial _{\mu }K^{+}\partial ^{\mu } {\bar{K}}^{0}\text { + h.c.}\right) . \end{aligned}$$

Appendix A.6: Lagrangian for $\eta _{S}^{E}$

The Lagrangian reads

A.6 $$\begin{aligned} \mathcal {L}_{\eta _{S}^{E}}&= -\frac{i}{\sqrt{2}}h_{3}^{\star }w_{K_{1} }Z_{K}\phi _{N}\,\eta _{S}^{E}\left( \partial _{\mu }{\bar{K}}^{0}K^{\star \mu 0}+\partial _{\mu }K^{-}K^{\star \mu +}\text { + h.c.}\right) \nonumber \\&\qquad \qquad +\,(h_{2}^{\star }-h_{3}^{\star })w_{a_{1}}^{2}Z_{\pi }^{3}\,\eta _{N}^{E}\left( \partial _{\mu }\eta _{N} \partial ^{\mu }\mathbf {\pi }\right) \cdot \mathbf {\pi }\nonumber \\&\qquad \qquad -\,\frac{1}{2\sqrt{2}}h_{2}^{\star }w_{a_{1}}w_{K_{1}}Z_{\pi }Z_{K}^{2} \,\eta _{S}^{E}\left( {\bar{K}}^{0}\partial _{\mu }K^{0}\partial ^{\mu }\pi ^{0}\right. \nonumber \\&\qquad \qquad \left. -\,\sqrt{2}{\bar{K}}^{0}\partial _{\mu }K^{+}\partial ^{\mu }\pi ^{-}\right. \nonumber \\&\qquad \qquad \left. -\,K^{-}\partial _{\mu }K^{+}\partial ^{\mu }\pi ^{0}-\sqrt{2} K^{-}\partial _{\mu }K^{0}\partial ^{\mu }\pi ^{+}\text { + h.c.}\right) \nonumber \\&\qquad \qquad +\,\frac{1}{\sqrt{2}}h_{3}^{\star }w_{K_{1}}^{2}Z_{\pi }Z_{K}^{2}\,\eta _{S} ^{E}\nonumber \\&\qquad \qquad \times \left( \pi ^{0}\partial _{\mu }{\bar{K}}^{0}\partial ^{\mu }K^{0}+\pi ^{0}\partial _{\mu }K^{-}\partial ^{\mu }K^{+}\right. \nonumber \\&\qquad \qquad \left. +\,\sqrt{2}\pi ^{-}\partial _{\mu } K^{+}\partial ^{\mu }{\bar{K}}^{0}\text { + h.c.}\right) . \end{aligned}$$

Note: the decay $\eta _{S}^{E}\rightarrow \eta _{S}\pi \pi$ ($\sim \kappa _{1}$, $h_{1}^{\star }$) is large-$N_{c}$ suppressed.

Appendix A.7: Lagrangian for $\pi ^{E}$

The Lagrangian reads (only $\pi ^{0E}$ included; decays of $\pi ^{\pm E}$ follow from isospin symmetry):

A.7 $$\begin{aligned}&\mathcal {L}_{\pi ^{E}} =-\,ih_{3}^{\star }w_{a_{1}}Z_{\pi }\phi _{N}\,\pi ^{0E}\left( \rho _{\mu }^{-}\partial ^{\mu }\pi ^{+}-\rho _{\mu }^{+}\partial ^{\mu }\pi ^{-}\right) \nonumber \\&\qquad \qquad +\,\frac{1}{4}\left( h_{2}^{\star }-2h_{3}^{\star }\right) w_{a_{1}}w_{K_{1} }Z_{\pi }Z_{K}^{2}\,\pi ^{0E}\partial _{\mu }\pi ^{0}\nonumber \\&\qquad \qquad \times \,\left( {\bar{K}}^{0} \partial ^{\mu }K^{0}+K^{-}\partial ^{\mu }K^{+}\text { + h.c.}\right) \nonumber \\&\qquad \qquad +\,\frac{1}{2}h_{2}^{\star }w_{K_{1}}^{2}Z_{\pi }Z_{K}^{2}\,\pi ^{0E}\pi ^{0}\left( \partial _{\mu }{\bar{K}}^{0}\partial ^{\mu }K^{0}+\partial _{\mu } K^{-}\partial ^{\mu }K^{+}\right) \nonumber \\&\qquad \qquad -\,\frac{1}{2\sqrt{2}}\left( h_{2}^{\star }+2h_{3}^{\star }\right) w_{a_{1} }w_{K_{1}}Z_{\pi }Z_{K}^{2}\,\pi ^{0E}\nonumber \\&\qquad \qquad \times \,\left[ \partial _{\mu }\pi ^{-}\left( {\bar{K}}^{0}\partial ^{\mu }K^{+}-K^{+}\partial ^{\mu }{\bar{K}}^{0}\right) \text { + h.c.}\right] \nonumber \\&\qquad \qquad +\,\frac{1}{2}(h_{2}^{\star }+h_{3}^{\star })w_{a_{1}}^{2}Z_{\pi }^{3}\,\pi ^{0E}\pi ^{0}(\partial _{\mu }\mathbf {\pi })^{2}\nonumber \\&\qquad \qquad -\,h_{3}^{\star }w_{a_{1}}^{2}Z_{\pi }^{3}\,\pi ^{0E}\partial _{\mu }\pi ^{0}\left( \mathbf {\pi }\cdot \partial ^{\mu } \mathbf {\pi }\right) . \end{aligned}$$

Appendix A.8: Lagrangian for $K^{E}$

The Lagrangian reads (only $K^{0E}$ included; decays of other $K^{E}$ components follow from isospin symmetry):

A.8 $$\begin{aligned}&\mathcal {L}_{K^{E}} =-\frac{i}{4}\left[ h_{2}^{\star }\left( \phi _{N} \varvec{-} \sqrt{2}\phi _{S}\right) \varvec{+} 2h_{3}^{\star }\phi _{N}\right] w_{K_{1}} Z_{K}\,K^{0E}\nonumber \\&\qquad \qquad \times \, \left( \omega _{N\mu }\partial ^{\mu }{\bar{K}}^{0}-\rho _{\mu } ^{0}\partial ^{\mu }{\bar{K}}^{0}+\sqrt{2}\rho _{\mu }^{+}\partial ^{\mu }K^{-}\right) \nonumber \\&\qquad \qquad -\,\frac{i}{4}\left[ h_{2}^{\star }\left( \phi _{N}-\sqrt{2}\phi _{S}\right) -2h_{3}^{\star }\phi _{N}\right] w_{a_{1}}Z_{\pi }\,K^{0E}\nonumber \\&\qquad \qquad \times \,\left( {\bar{K}}_{\mu }^{\star 0}\partial ^{\mu }\eta _{N}-{\bar{K}}_{\mu }^{\star 0}\partial ^{\mu }\pi ^{0}+\sqrt{2}K_{\mu }^{\star -}\partial ^{\mu }\pi ^{+}\right) \nonumber \\&\qquad \qquad -\,\frac{i}{2\sqrt{2}}\left[ h_{2}^{\star }\left( \phi _{N}-\sqrt{2}\phi _{S}\right) +2\sqrt{2}h_{3}^{\star }\phi _{S}\right] \nonumber \\&\qquad \qquad \times \, w_{f_{1S}}Z_{\eta _{S} }\,K^{0E}{\bar{K}}_{\mu }^{\star 0}\partial ^{\mu }\eta _{S}\nonumber \\&\qquad \qquad -\,\frac{1}{2}h_{2}^{\star }w_{a_{1}}^{2}Z_{\pi }^{2}Z_{K}\,K^{0E}\left( {\bar{K}}^{0}\partial _{\mu }\eta _{N}\partial ^{\mu }\pi ^{0}\right. \nonumber \\&\qquad \qquad \left. -\sqrt{2}K^{-} \partial _{\mu }\eta _{N}\partial ^{\mu }\pi ^{+}\right) \nonumber \\&\qquad \qquad -\,\frac{1}{4}(h_{2}^{\star }-2h_{3}^{\star })w_{a_{1}}w_{K_{1}}Z_{\pi } ^{2}Z_{K}\,K^{0E}\left( \pi ^{0}\partial _{\mu }\eta _{N}\partial ^{\mu }\bar{K}^{0}\right. \nonumber \\&\qquad \qquad \left. -\,\sqrt{2}\pi ^{+}\partial _{\mu }\eta _{N}\partial ^{\mu }K^{-}+\eta _{N}\partial _{\mu }\pi ^{0}\partial ^{\mu }{\bar{K}}^{0}\right. \nonumber \\&\qquad \qquad \left. -\,\sqrt{2}\eta _{N} \partial _{\mu }\pi ^{+}\partial ^{\mu }K^{-}\right) \nonumber \\&\qquad \qquad +\,\frac{1}{\sqrt{2}}h_{3}^{\star }w_{a_{1}}w_{f_{1S}}Z_{\pi }Z_{K}Z_{\eta _{S} }\,K^{0E}\nonumber \\&\qquad \qquad \times \, \left( {\bar{K}}^{0}\partial _{\mu }\eta _{S}\partial ^{\mu }\pi ^{0}\right. \nonumber \\&\qquad \qquad \left. -\,\sqrt{2}K^{-}\partial _{\mu }\eta _{S}\partial ^{\mu }\pi ^{+}\right) \nonumber \\&\qquad \qquad -\, \frac{1}{2\sqrt{2}}h_{2}^{\star }w_{K_{1}}w_{f_{1S}}Z_{\pi }Z_{K}Z_{\eta _{S}}\,K^{0E}\nonumber \\&\qquad \qquad \times \,\left( \pi ^{0}\partial _{\mu }\eta _{S}\partial ^{\mu }{\bar{K}} ^{0}-\sqrt{2}\pi ^{+}\partial _{\mu }\eta _{S}\partial ^{\mu }K^{-}\right) \nonumber \\&\qquad \qquad -\, \frac{1}{2\sqrt{2}}h_{2}^{\star }w_{a_{1}}w_{K_{1}}Z_{\pi }Z_{K}Z_{\eta _{S} }\,K^{0E}\nonumber \\&\qquad \qquad \times \,\left( \eta _{S}\partial _{\mu }\pi ^{0}\partial ^{\mu }{\bar{K}}^{0} -\sqrt{2}\eta _{S}\partial _{\mu }\pi ^{+}\partial ^{\mu }K^{-}\right) \nonumber \\&\qquad \qquad +\,\frac{1}{4}h_{2}^{\star }w_{a_{1}}^{2}Z_{\pi }^{2}Z_{K}\,K^{0E}{\bar{K}} ^{0}(\partial _{\mu }\mathbf {\pi })^{2}+\frac{1}{4}(h_{2}^{\star }-2h_{3}^{\star })\nonumber \\&\qquad \qquad \times \, w_{a_{1}}w_{K_{1}}Z_{\pi }^{2}Z_{K}\,K^{0E}\pi ^{0}\partial _{\mu }{\bar{K}} ^{0}\partial ^{\mu }\pi ^{0}\nonumber \\&\qquad \qquad -\, h_{3}^{\star }w_{a_{1}}w_{K_{1}}Z_{\pi }^{2}Z_{K}\,K^{0E}\pi ^{-} \partial _{\mu }{\bar{K}}^{0}\partial ^{\mu }\pi ^{+}+\frac{1}{2}h_{2}^{\star }\nonumber \\&\qquad \qquad \times \,w_{a_{1}}w_{K_{1}}Z_{\pi }^{2}Z_{K}\,K^{0E}\pi ^{+}\partial _{\mu }{\bar{K}} ^{0}\partial ^{\mu }\pi ^{-}\nonumber \\&\qquad \qquad +\,\frac{1}{2\sqrt{2}}(h_{2}^{\star }+2h_{3}^{\star })w_{a_{1}}w_{K_{1}}Z_{\pi }^{2}Z_{K}\,K^{0E}\nonumber \\&\qquad \qquad \times \, \left( \pi ^{+}\partial _{\mu }K^{-}\partial ^{\mu }\pi ^{0} -\pi ^{0}\partial _{\mu }K^{-}\partial ^{\mu }\pi ^{+}\right) . \end{aligned}$$

## Footnotes

1 There is a subtle point pertaining to the condensation of excited states in $\sigma$-type models: as discussed in Ref. [184], it can be in agreement with QCD constraints but may also, depending on parameter choice, spontaneously break parity in vacuum. Study of a model with condensation of the excited states would go beyond the current work. (It would additionally imply that the excited pseudoscalars also represent Goldstone bosons of QCD which is disputed in, e.g., Ref. [111].)

2 The $\eta (1405)$ resonance would then be a candidate for the pseudoscalar glueball [202].

3 However, there would be no mixing of pseudoscalar isosinglets $\eta _{N}^{E}$ and $\eta _{S}^{E}$ in the model even if all discarded parameters were considered. The reason is that there is no condensation of excited scalar states in Lagrangian (16).

4 A similar mixing scenario may (as a matter of principle) also exist in the case of the scalars discussed here. However, the amount of theoretical studies is significantly smaller here: for example, a glueball contribution to $f_0(1790)$ has been discussed in Refs. [210, 211] while – just as in our study – the same resonance was found to be compatible with an excited ${\bar{q}}q$ state in Ref. [102].
